# “On Water” Palladium Catalyzed Direct Arylation of 1*H*-Indazole and 1*H*-7-Azaindazole

**DOI:** 10.3390/molecules25122820

**Published:** 2020-06-18

**Authors:** Khadija Gambouz, Abdelmoula El Abbouchi, Sarah Nassiri, Franck Suzenet, Mostapha Bousmina, Mohamed Akssira, Gérald Guillaumet, Saïd El Kazzouli

**Affiliations:** 1 Faculty of Sciences and Technologies Mohammedia, University Hassan 2, URAC 22 FSTM University Hassan II—Casablanca, BP 146, Mohammedia 28800, Morocco; gambouzkhadija@gmail.com (K.G.); akssira.m@gmail.com (M.A.); 2 Institut de Chimie Organique et Analytique, University of Orléans, UMR CNRS 7311, BP 6759, CEDEX 2, 54067 Orléans, France; a.elabbouchi@ueuromed.org (A.E.A.); sarah.nassiri@femg.ueuromed.org (S.N.); franck.suzenet@univ-orleans.fr (F.S.); 3 Euromed Research Center, Euromed Institute of Technology, Euromed University of Fes (UEMF), Route de Meknès, Rond-point de Bensouda, Fes 30000, Morocco; m.bousmina@ueuromed.org

**Keywords:** indazole, azaindazole, arylation, green chemistry, C–H functionalization, water

## Abstract

The C3 direct arylation of 1*H*-indazole and 1*H*-7-azaindazole has been a significant challenge due to the lack of the reactivity at this position. In this paper, we describe a mild and an efficient synthesis of new series of C3-aryled 1*H*-indazoles and C3-aryled 1*H*-7-azaindazoles via a C3 direct arylation using water as solvent. On water, PPh_3_ was effective as a ligand along with a lower charge of the catalyst Pd(OAc)_2_ (5 mol%) at 100 °C, leading to C3-aryled 1*H*-indazoles or C3-aryled 1*H*-7-azaindazoles in moderate to good yields.

## 1. Introduction

In recent years, the C–H activation has risen as an increasingly powerful tool for molecular sciences with notable applications in organic synthesis [1,2,3,4,5]. This method has gained considerable recent momentum as a significantly environmentally and economically attractive alternative to classical cross-coupling such as Suzuki–Miyaura, Negishi, and Stille reactions [6,7,8,9,10]. With fewer steps and accessible reagents, complex organic molecules are nowadays easily accessible with C–H activation [11,12,13,14,15,16,17,18,19,20]. Nevertheless, this reaction usually requires high temperatures and organic solvents to achieve new C(sp^2^)–C(sp^2^) bonds [21]. For economic and environmental concerns, we are aiming at developing a new C–H direct arylation of 1*H*-indazole and 1*H*-7-azaindazole by using water as a green solvent. Water is nature’s primordial solvent to carry out synthesis [22]. It is known that water can increase the rate and affect the selectivity of a wide variety of organic reactions [23,24,25,26]. It was also found that on watery conditions, the heterogeneous mixture of substrates and catalyst can be very effective for direct arylation under mild conditions [27]. 

**Scheme 1:**
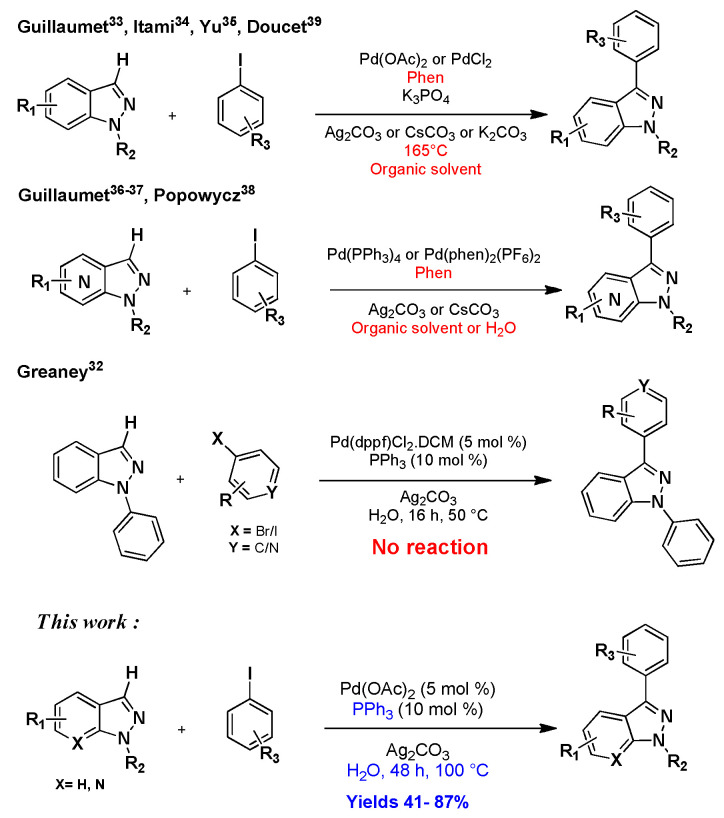
Reported methods on C3 arylation of 1*H*-indazoles and 1*H*-azaindazoles versus our new procedure.

1*H*-indazoles and 2*H*-indazoles have widespread utility as highly bioactive molecules [28]. For such reasons, various researchers were inspired to develop and optimize new methods for their synthesis and functionalization [29,30,31,32]. Recently, different research groups have investigated the C3 direct arylation of indazole, including our own. In 2012, our group [33] in parallel with the Itami group [34] developed, for the first time, conditions to realize the direct arylation of 1*H*-indazole series. The key of this success was the use of bidentate ligand (1,10-phenanthroline) with a high catalyst loading (10 to 20 mol%). Later, the reaction conditions have been optimized by the Yu group by reducing catalyst and ligand loading [35]. In this report, only one example of C3 arylation of 1*H*-7-azaindazole was reported using Pd(OAc)_2_ as a catalyst, and Phen as a ligand in toluene at 160 °C (Scheme 1). Very recently, our group [36,37] and later Popowycz’s [38] group reported independently that the C3 direct arylation of azaindazoles was feasible but using again 1,10-phenanthroline (Phen) as a crucial ligand in the case of 1*H* series for both 4-azaindazoles and 7-azaindazoles. In 2017, Doucet and his group reported a phosphine free C3 arylation of 2*H*-indazoles in dimethylacetamide DMA using 5 mol% of Pd(OAc)_2_ as catalyst at 150 °C [39]. We noticed in their report that the C3 direct arylation of 1*H*-indazole was feasible for the first time without the use of bidentate ligands by employing PdCl(C_3_H_5_)(dppb) as catalyst and KOAc as base in DMA at 150 °C. Nevertheless, the expected products were obtained in moderate to low yields, and the reaction was achieved in organic solvent. It is noteworthy that all the reported methods on C3 arylation of 1*H*-indazole discussed above were carried out in organic solvents and at high temperatures. In 2010, Greaney et al. [32] reported that 2-phenyl-2*H*-indazole could be directly arylated at the C3 position in high yields using aryl bromides as coupling partners on water in the presence of Pd(dppf)Cl_2_ (5 mol%) and Ph_3_P (10 mol%) as catalyst and ligand, respectively. Unfortunately, under these reaction conditions, the authors failed to achieve direct arylation on 1-phenyl-1*H*-indazole, yet they concluded that, in this case, the C3 position is non-reactive toward substitution. 

Herein, we reported a new path for the direct and selective C3 arylation of 1*H*-indazoles on water. We showed, for the first time, that a phosphine ligand (PPh_3_), contrary to the bidentate (Phen) ligand used in the case of organic solvent, is crucial to achieve the arylation reaction (Scheme 1). We are striving also to report the first examples of “on water” C3 arylation of 1*H*-7-azaindazoles. We showed as well that a low charge of the catalyst (5 mol%) and ligand (10 mol%) leads to desired products in acceptable to good yields. 

It is noticed that Knochel et al. used 3-zincated indazoles which undergo palladium-catalyzed Negishi cross-couplings to give the C3 substituted indazole derivatives [40], while, Burton et al. described a regioselective iridium-catalyzed C3 borylation of 1*H*-indazoles, followed by subsequent Suzuki–Miyaura coupling with aryl chlorides [41]. Compared to the methodology described in our manuscript, the two methodologies cited above are more difficult to implement and also less economical.

## 2. Results

Firstly, we tested Greaney’s reaction conditions [Ag_2_CO_3_ (1 equivalent), PPh_3_ (10 mol%), Pd(dppf)Cl_2_·DCM (5 mol%), 4-iodotoluene (1.1 equivalent), water at 50 °C for 16 h] [32] on 1-methyl-1*H*-indazole **1**. The arylation reaction did not occur, and only starting material **1** was recovered (Table 1, entry 1).

Then, in the presence of palladium(II)acetate and 1,10-phenanthroline as a bidentate ligand, again no reaction was observed, and only starting material **1** was recovered (Table 1, entry 2). Changing the type of the ligand to triphenylphosphine, a monodentate ligand, which may form a stronger coordination with Pd(II) centers, led to the desired arylated product **1a** in 40% yield (Table 1, entry 3). Encouraged by this result, we decided to improve the reaction’s conversion by screening different conditions. At first, we tried to increase the temperature and to change the heating system with our efforts leading to no avail (Table 1, entries 4–6). Water/ethanol solution was also used to increase the solubility, but only a decrease of the reaction yield (17%) was observed (Table 1, entry 7). Using (2 equiv.) of iodotoluene slightly improved the reaction yield (entry 8). Luckily, increasing the amount of iodotoluene to (3 equiv.) furnished **1a** to 86% isolated yield and with a total conversion (Table 1, entry 9). Furthermore, we succeeded in reducing the loading of both palladium and PPh_3_ ligand to 5 and 10% respectively (Table 1, entry 11). When the reaction was run for 24 h instead of 48 h, the yield dramatically decreased (Table 1, entry 12). Notably, the reaction temperature can be reduced to 100 °C without affecting the reaction yield (Table 1, entry 13). Using Phen instead of Ph_3_P as a ligand, under the optimized reaction conditions, led to a total loss of the reactivity (Table 1, entry 14). The reaction under reflux of water in an open flask was not total and 10% of starting material **1** was recovered (Table 1, entry 15). In addition, when 4-iodotoluene was replaced by 4-bromotoluene, the arylation did not occur, and only starting material **1** was recovered (Table 1, entry 16).

**Scheme 2:**
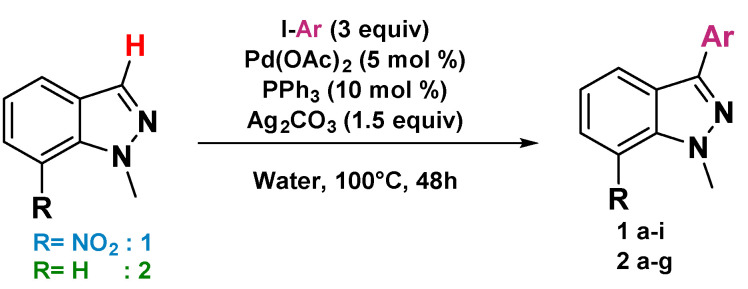

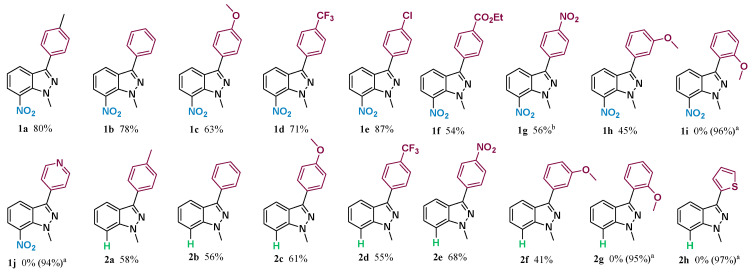
Scope and limitation of the ‘’on water’’ direct arylation of 1-methyl-7-nitro-1*H*-indazole and 1-methyl-1*H*-indazole. ^a^ Percent of recovered starting material, ^b^ 72 h.

The scope and limitation of the C3 arylation reaction of various substituted indazoles **1** and **2** were examined (their preparation is described in the Supporting Information). We used various iodoaryl derivatives carrying electron-donating or electron-withdrawing groups (such as methoxy, chlorine, ester, nitro, trifluoromethyl, or methyl groups) under the most optimal reaction conditions [5 mol% Pd(OAc)_2_ as the catalyst, 10% PPh_3_ as the ligand, and Ag_2_CO_3_ as the base in water at 100 °C (Scheme 2)]. The results showed that the expected C3-arylated products **1a**–**g** were regioselectivity obtained. First, using indazole **1** with 4-iodotoluene gave **1a** in 80% yield (Scheme 2). Afterwards, the coupling of **1** with various aryl iodides was studied. Thus, the reactions with iodobenzene, 4-iodoanisole, 4-iodobenzotrifluoride, 1-chloro-4-iodobenzene, ethyl 4-iodobenzoate, and 1-iodo-4-nitrobenzene afforded **1b**–**g** in 54–87% yields (Scheme 2). A moderate yield was observed when 3-iodoanisole was used. In this case, the desired product **1h** was isolated in 45% yield. No reaction was observed when indazole **1** was treated by 2-iodoanisole presumably as a result of its steric hindrance (methoxy group at the 2-position of the aromatic ring) and 4-iodopyridine (Scheme 2). It is noticeable that iodoaryl, containing electron-donating groups, was crucial for the achievement of C3 arylation reactions in good reaction yields (63–87%). In our second attempt, we used indazole **2** in order to see if the nitro group on position C7 affects the arylation regioselectivity. The treatment of indazole **2** with various substituted aryl iodides as coupling partners showed that the nature of the substituents (electron-donating or electron-withdrawing groups) did not affect the reaction yields or regioselectivity (compounds **2a**–**f**). Thus, the desired products **2a**–**f** were obtained in yields ranging between 41 and 68%. As described in the previous results, we also observed no arylation when indazole **2** was treated by 2-iodoanisole and hetero-aryl iodide as coupling partners (compounds **2g**–**h**) (Scheme 2).

**Scheme 3:**
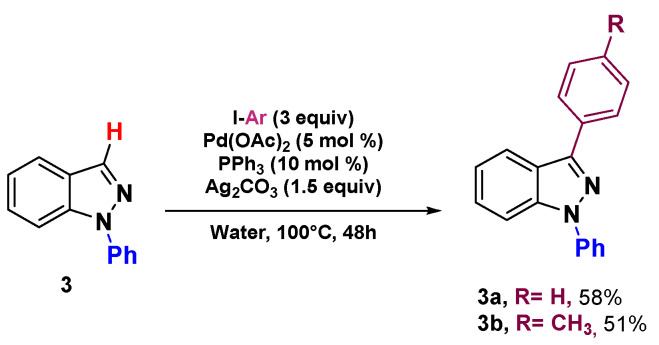
Direct arylation of 1-phenyl 1*H*-indazole **3**.

Finally, we decided to run the reaction under our reaction conditions using the starting material **3** previously used without success by Greaney and his group [32]. As expected, we succeeded in achieving the desired arylated products **3a** and **3b** in 58 and 51% yield, respectively (Scheme 3).

**Scheme 4:**
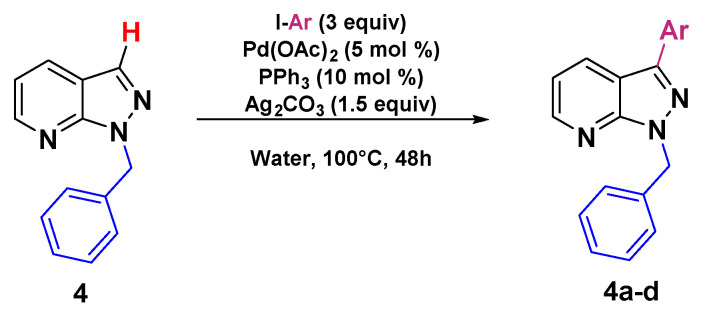

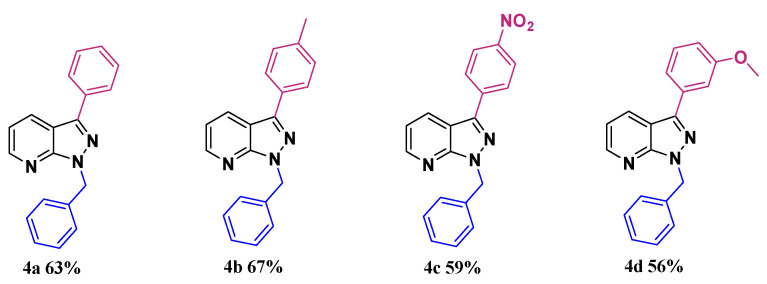
Direct arylation "on water" of pyrazolo [3,4-b] pyridine.

After succeeding in developing a “greener” pathway to synthesize C3-arylated 1*H*-indazole, we extended the methodology to prepare C3-arylated pyrazolo[3,4-*b*]pyridine, also known as azaindazole, which is an 1*H*-indazole isostere and is often used as a key pharmacophore in drug design [42,43,44,45,46,47,48,49,50,51,52,53,54,55,56,57]. Despite the enormous biological interest of C3-arylated pyrazolo [3,4-*b*]pyridines, only one work has been reported by Lavard and Popowycz [38], addressing the functionalization of this motif via direct arylation. Although this reported method is efficient, it is relatively energy-wasting, using heating at 160 °C for a long period of 3 days and organic solvent. For this reason, we decided to test our conditions developed to prepare C3-arylated pyrazolo[3,4-*b*]pyridine using the starting material **4** containing a benzyl group (the preparation of starting material **4** is described in the Supporting Information) with various aryl iodides as arylating partners. As expected, this procedure also showed a very high tolerance to various substituents on the aryl rings and the arylated products **4b**–**d** were isolated with yields ranging between 56 and 67% (Scheme 4).

## 3. Materials and Methods 

### 3.1. Instrumenttion

The reactions were monitored by thin-layer chromatography (TLC) analysis using silica gel (60 F254) plates. Compounds were visualized by UV irradiation. Flash column chromatography was performed on silica gel 60 (230–400.13 mesh, 0.040, 0.063 mm). Melting points (m_p_ [°C]) were taken on samples in open capillary tubes and are uncorrected. The infrared spectra of compounds were recorded at room temperature on a Thermo Scientific Nicolet IS50 FT-IR. ^1^H and ^13^C NMR spectra were recorded on a Bruker Avance II 400 MHz (^13^C, 100 MHz) or on a Bruker Avance DPX 250 MHz (^13^C, 62.9 MHz). Chemical shifts are given in parts per million from tetramethylsilane (TMS) as internal standard. The multiplicities of the spectra are reported as follows: singlet (s), doublet (d), triplet (t), quartet (q), and multiplet (m). Coupling constants (*J*) are reported in hertz (Hz). High-resolution mass spectra (HRMS) were performed on a Maxis Bruker 4G.

### 3.2. Preparation of Starting Compounds **1**–**4**

*N*-Methylation of indazole [33]: the indazole (1 g, 1 equivalent) was dissolved in acetone (10 mL) at 0 °C in a 50 mL flask. KOH (3 equivalent) was added, and then CH_3_I (1.5 equivalent) was added dropwise. The reaction mixture was filtered and separated by flash chromatography on silica gel (75% yield).

*N*-Arylation of indazole [42]: iodobenzene (204 mg, 1 mmol) and dimethyl sulfoxide 1 mL was added to the mixture of 1*H*-indazole (141.7 mg, 1.2 mmol), KOH (67.3 mg, 1.2 mmol), and copper iodide (I) (19.1 mg, 0.1 mmol), and the reaction was for 12 h at 120 °C. After the completion of the reaction, cooled to room temperature, 2 mL of water and ethyl acetate 2 mL was added, and liquid separation was done. 1-phenyl indazole was obtained as a main component of the organic layer (80% yield).

*N*-Benzylation of 1*H*-pyrazolo[3,4-*b*]pyridine [33]: 1*H*-pyrazolo[3,4-*b*]pyridine (1 g, 8.39 mmol, 1.00 equivalent) was dissolved in acetone (10 mL) at 0 °C in a 50 mL flask, along with KOH (1.41 g, 25.19 mmol, 3.00 equivalent). After few minutes of stirring, benzyl chloride (1.59 g, 12.59 mmol, 1.50 equivalent) was added dropwise. The reaction mixture was filtered, and the two isomers N1 and N2 were separated by flash chromatography on silica gel (N1: 50%/N2: 45%).

### 3.3. General Experimental Procedure for the Synthesis of Products **1a**–**h, 2a**–**f**, **3a**–**b** and **4a**–**d**

A 5 mL sealed tube was charged with 1-methyl-7-nitro-1*H*-indazole **1**, 1-methyl-1*H*-indazole **2**, 1-phenyl-1*H*-indazole **3**, or 1-benzyl-1*H*-pyrazolo[3,4-*b*]pyridine **4** (1.0 equivalent), iodoaryl (3.0 equivalent), Pd(OAc)_2_ (0.05 equivalent), PPh_3_ (0.1 equivalent) and Ag_2_CO_3_ (1.5 equivalent). The mixture of solids was stirred for a few seconds to ensure all solids were well mixed. Then, 3 mL of distilled water was added, the mixture was degassed for few minutes, and the vial was covered with a serum cap. Then, the vial and its contents were heated and stirred at 100 °C for 24 h. After it was cooled to room temperature, the mixture was filtered through celite, and the organic phase was extracted three times with ethyl acetate, dried over magnesium sulfate, and then concentrated under reduced pressure. The residue was purified by flash chromatography to provide the desired products.

*1-Methyl-7-nitro-3-(p-tolyl)-1H-indazole***1a:** Yield: 80%; yellow solid; m_p_ = 154–156 °C; ^1^H NMR (400 MHz, CDCl_3_) δ 8.15 (dd, *J* = 8.1, 1.0 Hz, 1H), 8.06 (dd, *J*= 8.1, 1.0 Hz, 1H), 7.66 (d, *J* = 7.6 Hz, 2H), 7.23 (d, *J* = 7.6 Hz, 2H), 7.20–7.12 (m, 1H), 4.18 (s, 3H), 2.35 (s, 3H); ^13^C NMR (101 MHz, CDCl_3_) δ 145.5, 138.9, 135.3, 132.6, 129.8 (2 × C–H), 129.1, 128.3, 127.9 (2 × C–H), 126.9, 124.7, 119.9, 40.9, 21.5; HRMS (*m*/*z*) [M + H]^+^ calculated mass for C_15_H_14_N_3_O_2_, 268.1081, mass found 268.1079; IR (neat) ῠ = 1303, 1325, 1471, 1509, 2918, 3099 cm^−1^.

*1-Methyl-7-nitro-3-phenyl-1H-indazole***1b:** Data for compound **1b** were previously reported [11].

*3-(4-Methoxyphenyl)-1-methyl-7-nitro-1H-indazole***1c:** Yield: 63%; yellow solid; m_p_ = 182–184 °C; ^1^H NMR (400 MHz, CDCl_3_) δ 8.27 (dd, *J* = 8.1, 1.0 Hz, 1H), 8.17 (dd, *J* = 8.1, 1.0 Hz, 1H), 7.84 (d, *J* = 8.7 Hz, 2H), 7.31 (t, *J* = 7.9 Hz, 1H), 7.11 (d, *J* = 8.7 Hz, 2H), 4.31 (s, 3H), 3.93 (s, 3H); ^13^C NMR (101 MHz, CDCl_3_) δ 160.3, 145.3, 139.2, 132.7, 129.3 (2 × C–H), 128.3, 126.9, 124.7, 124.5, 119.9, 114.6 (2 × C–H), 55.5, 29.8; HRMS (*m*/*z*) [M + H]^+^ calculated mass for C_15_H_14_N_3_O_3_, 284.1030, mass found 284.1030; IR (neat) ῠ = 1321, 1361, 1514, 1530, 1612, 2849, 2919 cm^−1^.

*1-Methyl-7-nitro-3-(4-(trifluoromethyl)phenyl)-1H-indazole***1d:** Yield: 55%; yellow solid; m_p_ = 201–203 °C; ^1^H NMR (400 MHz, CDCl_3_) δ 8.25 (dd, *J* = 8.1, 1.0 Hz, 1H), 8.15 (dd, *J* = 8.1, 1.0 Hz, 1H), 8.07–7.95 (m, 2H), 7.85–7.73 (m, 2H), 7.32 (t, *J* = 7.9 Hz, 1H), 4.30 (s, 3H); ^13^C NMR (101 MHz, CDCl_3_) δ 143.8, 139.4, 135.6, 132.5, 131.0 (q, *J_Cq-F_* = 32.3 Hz, C_q_-F), 128.1 (2 × C–H), 127.6, 126.5, 126.1 (q, ^3^*J*_CHAr-F_ = 3.8 Hz, 2C, CHAr), 125.0 (q, ^1^*J_C-F_* = 272.1 Hz, CF_3_), 124.8, 120.1, 29.8;HRMS (*m*/*z*) [M + H]^+^ calculated mass for C_15_H_11_F_3_N_3_O_2_, 322.0798 mass found 322.0796; IR (neat) ῠ = 1275, 1329, 1346, 1498, 1540, 3020 cm^−1^.

*3-(4-Chlorophenyl)-1-methyl-7-nitro-1H-indazole***1e:** Yield: 87%; yellow solid; m_p_ = 172–173 °C; ^1^H NMR (400 MHz, CDCl_3_) δ 8.22 (dd, *J* = 8.1, 1.0 Hz, 1H), 8.15 (dd, *J* = 8.1, 1.0 Hz, 1H), 7.82 (d, *J* = 8.5 Hz, 2H), 7.51 (d, *J* = 8.5 Hz, 2H), 7.34–7.23 (m, 1H).4.29 (s, 3H); ^13^C NMR (101 MHz, CDCl_3_) δ 144.2, 135.5, 134.9, 132.7, 130.5, 129.4 (2 × C–H), 129.2 (2 × C–H), 127.8, 126.6, 124.8, 120.4, 29.8; HRMS (*m*/*z*) [M + H]^+^ calculated mass for C_14_H_11_ClN_3_O_2_, 288.0534, mass found 288.0534; IR (neat) ῠ =763, 1336, 1358, 1488, 1510, 3018 cm^−1^.

*Ethyl 4-(1-methyl-7-nitro-1H-indazol-3-yl)benzoate***1f:** Yield: 54%; yellow solid; m_p_ = 206–208 °C; ^1^H NMR (400 MHz, CDCl_3_) δ 8.27 (dd, *J* = 8.1, 1.0 Hz, 1H), 8.20 (m, 2H), 8.17 (d, *J* = 8.1 Hz, 1H), 7.98 (m, 2H), 7.32 (t, *J* = 7.9 Hz, 1H), 4.43 (q, *J* = 7.1 Hz, 2H), 4.31 (s, 3H), 1.44 (t, *J* = 7.1 Hz, 3H); ^13^C NMR (101 MHz, CDCl_3_) δ 166.3, 144.2, 136.3, 135.6, 132.7, 130.6, 130.3 (2 × C–H), 127.9, 127.7 (2 × C–H), 126.7, 124.8, 120.6, 61.3, 41.2, 14.5; HRMS (*m*/*z*) [M + H]^+^ calculated mass for C_17_H_16_N_3_O_4_, 326.1135, mass found 326.1135; IR (neat) ῠ = 1100, 1259, 1320, 1365, 1514, 1708, 2850, 2919, 2986, 3104 cm^−1^.

*1-Methyl-7-nitro-3-(4-nitrophenyl)-1H-indazole***1g:** Yield: 56%; yellow solid; m_p_ = 242–244 °C; ^1^H NMR (400 MHz, CDCl_3_) δ 8.40 (d, *J* = 8.9 Hz, 2H), 8.28 (dd, *J* = 8.1, 1.0 Hz, 1H), 8.18 (dd, *J* = 8.1, 1.0 Hz, 1H), 8.10 (d, *J* = 8.9 Hz, 2H), 7.37 (t, *J* = 7.9 Hz, 1H), 4.32 (s, 3H); ^13^C NMR (101 MHz, CDCl_3_) δ 148.5, 142.8, 138.5 (2 × C–H), 132.8, 132.2, 128.4 (2 × C–H), 127.4, 124.9, 124.4 (2 × C–H), 121.2, 29.8; HRMS (*m*/*z*) [M + H]^+^ calculated mass for C_14_H_11_N_4_O_4_, 299.0775 mass found 299.0774; IR (neat) ῠ = 1325, 1345, 1455, 1518, 3076, 1601 cm^−1^.

*3-(3-Methoxyphenyl)-1-methyl-7-nitro-1H-indazole***1h:** Yield: 45%; yellow solid; m_p_ = 164–166°C; ^1^H NMR (400 MHz, CDCl_3_) δ 8.27 (dd, *J* = 8.1, 1.0 Hz, 1H), 8.14 (dd, *J* = 8.1, 1.0 Hz, 1H), 7.51–7.37 (m, 3H), 7.28 (d, *J* = 7.9 Hz, 1H), 7.00 (m, 1H), 4.29 (s, 3H), 3.91 (s, 3H); ^13^C NMR (101 MHz, CDCl_3_) δ 160.2, 145.3, 133.3, 132.7, 130.2, 128.7,128.2, 126.8, 124.7, 120.5, 120.1, 114.7, 113.4, 55.5, 41.0; HRMS (*m*/*z*) [M + H]^+^ calculated mass for C_15_H_14_N_3_O_3_, 284.1030, mass found 284.1029; IR (neat) ῠ = 1250, 1319, 1472, 1514, 1596, 2850, 2920 cm^−1^.

*1-Methyl-3-(p-tolyl)-1H-indazole***2a:** Data for compound **2a** were previously reported [35].

*1-Methyl-3-phenyl-1H-indazole***2b:** Data for compound **2b** were previously reported [35].

*3-(4-Methoxyphenyl)-1-methyl-1H-indazole***2c:** Data for compound **2c** were previously reported [35].

*1-Methyl-3-(4-(trifluoromethyl)phenyl)-1H-indazole***2d:** Data for compound **2d** were previously reported [35].

*1-Methyl-3-(4-nitrophenyl)-1H-indazole***2e:** Yield: 68%; white solid; m_p_ = 209–211 °C; ^1^H NMR (400 MHz, CDCl_3_) δ 8.37 (d, *J* = 8.8 Hz, 2H), 8.18 (d, *J* = 8.8 Hz, 2H), 8.03 (dd, *J* = 8.1, 1.1 Hz, 1H), 7.48 (d, *J* = 3.4 Hz, 2H), 7.30 (dt, *J* = 8.1, 3.4 Hz, 1H), 4.17 (s, 3H); ^13^C NMR (101 MHz, CDCl_3_) δ 147.1, 141.7, 141.2, 140.4, 127.5 (2 × C–H), 126.8, 124.3 (2 × C–H), 122.1, 121.7, 120.9, 109.8, 29.8; HRMS (*m*/*z*) [M + H]^+^ calculated mass forC_14_H_12_N_3_O_2_, 254.0924, mass found 254.0922; IR (neat) ῠ = 2931, 1543, 1486, 1352, 1347, 1260, 780 cm^−1^.

*3-(3-Methoxyphenyl)-1-methyl-1H-indazole***2f**: Yield: 41%; white solid; m_p_ = 112–113 °C; ^1^H NMR (400 MHz, CDCl_3_) δ 8.04 (d, *J* = 8.1 Hz, 1H), 7.57 (dd, *J* = 7.4, 1.4 Hz, 1H), 7.54 (d, *J* = 1.4 Hz, 1H), 7.49–7.37 (m, 3H), 7.22 (ddd, *J* = 8.1, 5.1, 2.7 Hz, 1H), 6.96 (d, *J* = 5.8 Hz, 1H), 4.13 (s, 3H), 3.91 (s, 3H); ^13^C NMR (101 MHz, CDCl_3_) δ 160.1, 143.6, 141.5, 135.1, 129.9, 126.3, 121.7, 121.4, 121.0, 120.0, 113.9, 112.6, 109.3, 55.4, 29.8; HRMS (*m*/*z*) [M + H]^+^ calculated mass for C_15_H_15_N_2_O, 239.1179 mass found 239.1176; IR (neat) ῠ =2931, 1486, 1347, 1289, 1260, 780 cm^−1^.

*1,3-Diphenyl-1H-indazole***3a:** Data for compound **3a** were previously reported [34].

*1-Phenyl-3-(p-tolyl)-1H-indazole***3b:** Data for compound **3b** were previously reported [34].

*1-Benzyl-3-phenyl-1H-pyrazolo[3,4-b]pyridine***4a:** Yield: 63%; white solid; m_p_ = 133–135 °C; ^1^H NMR (400 MHz, CDCl_3_) δ 8.60 (dd, *J* = 4.5, 1.5 Hz, 1H), 8.35 (dd, *J* = 8.1, 1.5 Hz, 1H), 8.04–7.95 (m, 2H), 7.52 (dd, *J* = 8.3, 6.8 Hz, 2H), 7.46–7.40 (m, 3H), 7.35–7.25 (m, 3H), 7.18 (dd, *J* = 8.1, 4.5 Hz, 1H), 5.82 (s, 2H); ^13^C NMR (101 MHz, CDCl_3_) δ 151.45, 148.90, 143.11, 137.24, 133.33, 130.50, 128.99 (2 × C–H), 128.65 (2 × C–H), 128.38, 127.98 (2 × C–H), 127.72 (2 × C–H), 127.22, 117.20, 113.77, 50.87, 14.25; HRMS (*m*/*z*) [M + H]^+^ calculated mass for C_19_H_17_N_3_, 286.1339, mass found 286.1338; IR (neat) ῠ = 3050, 3031, 1610, 1330 cm^−1^.

*1-Benzyl-3-(p-tolyl)-1H-pyrazolo[3,4-b]pyridine***4b:** Yield: 67%; Yellow solid; m_p_ = 85–90 °C; ^1^H NMR (400 MHz, CDCl_3_) δ 8.59 (dd, *J* = 4.5, 1.5 Hz, 1H), 8.34 (dd, *J* = 8.1, 1.5 Hz, 1H), 7.98–7.79 (m, 2H), 7.45–7.38 (m, 2H), 7.35–7.24 (m, 5H), 7.18 (dd, *J* = 8.1, 4.5 Hz, 1H), 5.80 (s, 2H), 2.44 (s, 3H); ^13^C NMR (101 MHz, CDCl_3_) δ 151.49, 148.87, 143.25, 138.32, 137.34, 130.59, 130.54, 129.72 (2 × C–H), 128.66 (2 × C–H), 127.98 (2 × C–H), 127.70, 127.14 (2 × C–H), 117.09, 113.81, 50.84, 21.48; HRMS (*m*/*z*) [M + H]^+^ calculated mass for C_20_H_18_N_3_, 300.1495, mass found 300.1499; IR (neat) ῠ = 2922, 2852, 1735, 1465, 772, 511 cm^−1^.

*1-Benzyl-3-(4-nitrophenyl)-1H-pyrazolo[3,4-b]pyridine***4c:** Yield: 59%; Yellow solid; m_p_ = 157–162 °C; ^1^H NMR (400 MHz, CDCl_3_) δ 8.66 (dd, *J* = 4.5, 1.5 Hz, 1H), 8.42–8.32 (m, 3H), 8.17 (d, *J* = 8.8 Hz, 2H), 7.45 (dd, *J* = 6.7, 1.7 Hz, 2H), 7.36–7.27 (m, 4H), 5.83 (s, 2H); ^13^C NMR (101 MHz, CDCl_3_) δ 151.50, 149.39, 147.38, 140.52, 139.71, 136.70, 130.04, 128.79 (2 × C–H), 128.17 (2 × C–H), 128.03, 127.39 (2 × C–H), 124.35 (2 × C–H), 118.14, 113.76, 51.23; HRMS (*m*/*z*) [M + H]^+^ calculated mass for C_19_H_15_N_4_O_2_, 331.1190, mass found 331.1191; IR (neat) ῠ = 2923, 2852, 1516, 1342, 771 cm^−1^.

*1-Benzyl-3-(3-methoxyphenyl)-1H-pyrazolo[3,4-b]pyridine***4d:** Yield: 56%; Yellow oil; ^1^H NMR (400 MHz, CDCl_3_) δ 8.58 (dd, *J* = 4.5, 1.5 Hz, 1H), 8.35 (dd, *J* = 8.1, 1.5 Hz, 1H), 7.58–7.48 (m, 2H), 7.46–7.36 (m, 3H), 7.30 (t, *J* = 7.2 Hz, 2H), 7.19 (dd, *J* = 8.1, 4.5 Hz, 1H), 6.96 (ddd, *J* = 8.2, 2.6, 1.0 Hz, 1H), 5.79 (s, 2H), 3.90 (s, 3H); ^13^C NMR (101 MHz, CDCl_3_) δ 160.19, 151.51, 148.99, 143.02, 137.26, 134.68, 130.58, 130.06, 128.70 (2 × C–H), 128.01 (2 × C–H), 127.76, 119.78, 117.30, 114.24, 113.87, 112.63, 55.55, 50.92; HRMS (*m*/*z*) [M + H]^+^ calculated mass for C_20_H_18_N_3_O, 316.1444, mass found 316.1443; IR (neat) ῠ = 2936, 2805, 1601, 1280 cm^−1^.

## 4. Conclusions

In summary, we have developed a new palladium catalyzed direct C3 arylation of 1*H*-indazole using a low charge of 5 mol% of Pd(OAc)_2_ along with 10 mol % of PPh_3_ on water as solvent at 100 °C. This new procedure afforded a board of arylated indazoles differently substituted at the C3 position in moderate to good yields. The application of this protocol on 1*H* 7-azaindazole was fruitful, and four examples of C3-arylated pyrazolo[3,4-*b*]pyridine were successfully reported. 

## Figures and Tables

**Table 1:** Optimization of the ‘’on water’’ direct arylation of 1-methyl-7-nitro-1*H*-indazole.

Entry	X	X (equiv)	Cat (equiv)	Ligand (equiv)	T °C	Time (h)	Solvent	Yield (%)
1	I	1.1	5% Pd(dppf)Cl_2_·DCM	10% PPh_3_	50	16	Water	0 ^[a]^ (85) ^[b]^
2	I	1.1	20% Pd(OAc)_2_	40% Phen	70	48	Water	0(80)
3	I	1.1	20% Pd(OAc)_2_	40% PPh_3_	70	48	Water	40 ^[c]^ (32)
4	I	1.1	20% Pd(OAc)_2_	40% PPh_3_	Reflux	48	Water	37 (29)
5	I	1.1	20% Pd(OAc)_2_	40% PPh_3_	Sealed tube 140 °C	48	Water	45 (28)
6	I	1.1	20% Pd(OAc)_2_	40% PPh_3_	Sealed tube 140 °C	48	Water	47 ^[d]^ (26)
7	I	1.1	20% Pd(OAc)_2_	40% PPh_3_	Sealed tube 140 °C	48	Water/EtOH 1:1	17(25)
8	I	2	20% Pd(OAc)_2_	40% PPh_3_	Sealed tube 140 °C	48	Water	50 (21)
9	I	3	20% Pd(OAc)_2_	40% PPh_3_	Sealed tube 140 °C	48	Water	86 (0)
10	I	3	10% Pd(OAc)_2_	10% PPh_3_	Sealed tube 140 °C	48	Water	84 (0)
11	I	3	5% Pd(OAc)_2_	10% PPh_3_	Sealed tube 140 °C	48	Water	81 (traces)
12	I	3	5% Pd(OAc)_2_	10% PPh_3_	Sealed tube 140 °C	24	Water	37 (58)
13	I	3	5% Pd(OAc)_2_	10% PPh_3_	Sealed tube 100 °C	48	Water	80 (traces)
14	I	3	5% Pd(OAc)_2_	10% Phen	Sealed tube 100 °C	48	Water	Traces (83)
15	I	3	5% Pd(OAc)_2_	10% PPh_3_	Reflux	48	Water	76 (10)
16	Br	3	5% Pd(OAc)_2_	10% PPh_3_	Sealed tube 100 °C	48	Water	0 (98)

^[a]^ Greaney’s conditions, ^[b]^ Percent of recovered starting material, ^[c]^ Yield of isolated product **2a**, ^[d]^ 10% Pd(OAc)_2_ and 20% PPh_3_ were used and after 24 h the same quantity of Pd(OAc)_2_ and PPh_3_ were added.

## Supplementary Materials

The following are available online, Spectral Data of All the Synthesized Products, NMR Spectra of All the Products.
