# Supplementary material for: “On Water” Palladium Catalyzed Direct Arylation of 1H-Indazole and 1H-7-Azaindazole
Source: Molecules. 2020 Jun 18;25(12):2820. doi: 10.3390/molecules25122820 (PMC7356112; doi:10.3390/molecules25122820)
Supplement: Supplementary file 1 [file molecules-25-02820-s001.pdf]

Supporting Information

"On Water" Palladium Catalyzed Direct Arylation of 1*H*-Indazole and 1*H*-7-Azaindazole

Khadija Gambouz [1,2,3,‡], Abdelmoula El Abbouchi [2,3,‡], Sarah Nassiri [2,3], Franck Suzenet [2], Mostapha Bousmina [3], Mohamed Akssira [1], Gérald Guillaumet [2,3,*] and Saïd El Kazzouli [3,*]

[1] Faculty of Sciences and Technologies Mohammedia, University Hassan 2, URAC 22 FSTM University Hassan II – Casablanca, BP 146, 28800 Mohammedia, Morocco; gambouzkhadija@gmail.com (K.G.); akssira.m@gmail.com (M.A.)

[2] Institut de Chimie Organique et Analytique, University of Orléans, UMR CNRS 7311, BP 6759, Orléans cedex 2 54067, France; a.elabbouchi@ueuromed.org (A.E.A.); sarah.nassiri@femg.ueuromed.org (S.N.); franck.suzenet@univ-orleans.fr (F.S.)

[3] Euromed Research Center, Euromed Institute of Technology, Euromed University of Fes (UEMF), Route de Meknès, Rond-point de Bensouda, 30000, Fes, Morocco; m.bousmina@ueuromed.org

‡ Both authors contributed equally to this manuscript

* Correspondence: gerald.guillaumet@univ-orleans.fr. (G.G.) and s.elkazzouli@ueuromed.org (S.E), Tel.: +212 661299565 (MO)

Contents

NMR Spectra of All the Products:... **2**

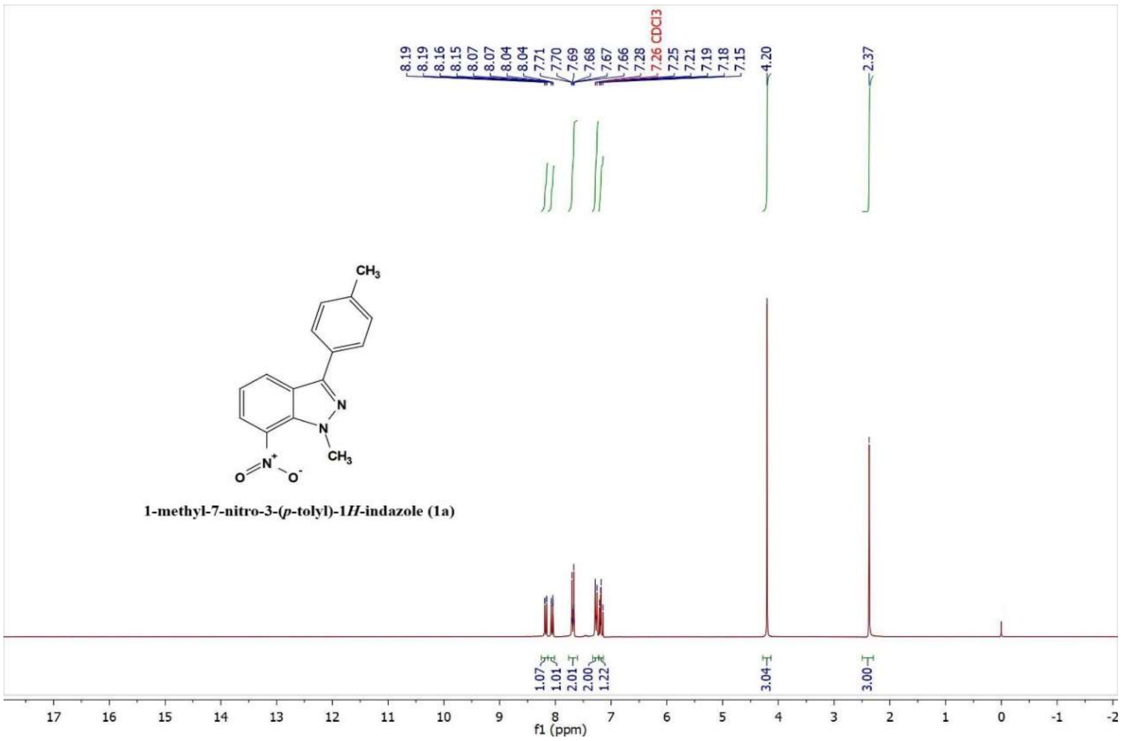

Figure 1 *: ^{1}H NMR spectrum (400 MHz, CDCl₃) of compound 1a*

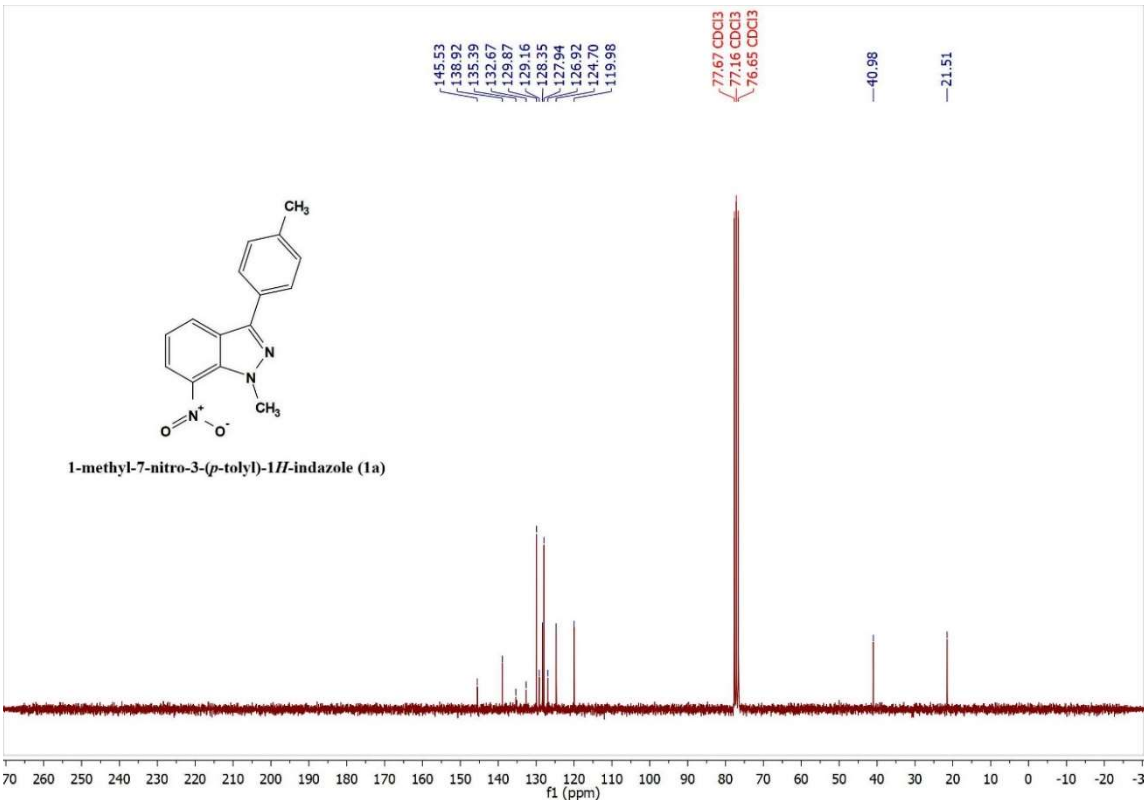

Figure 2 : *^{13}C NMR spectrum (101 MHz, CDCl₃) of compound 1a*

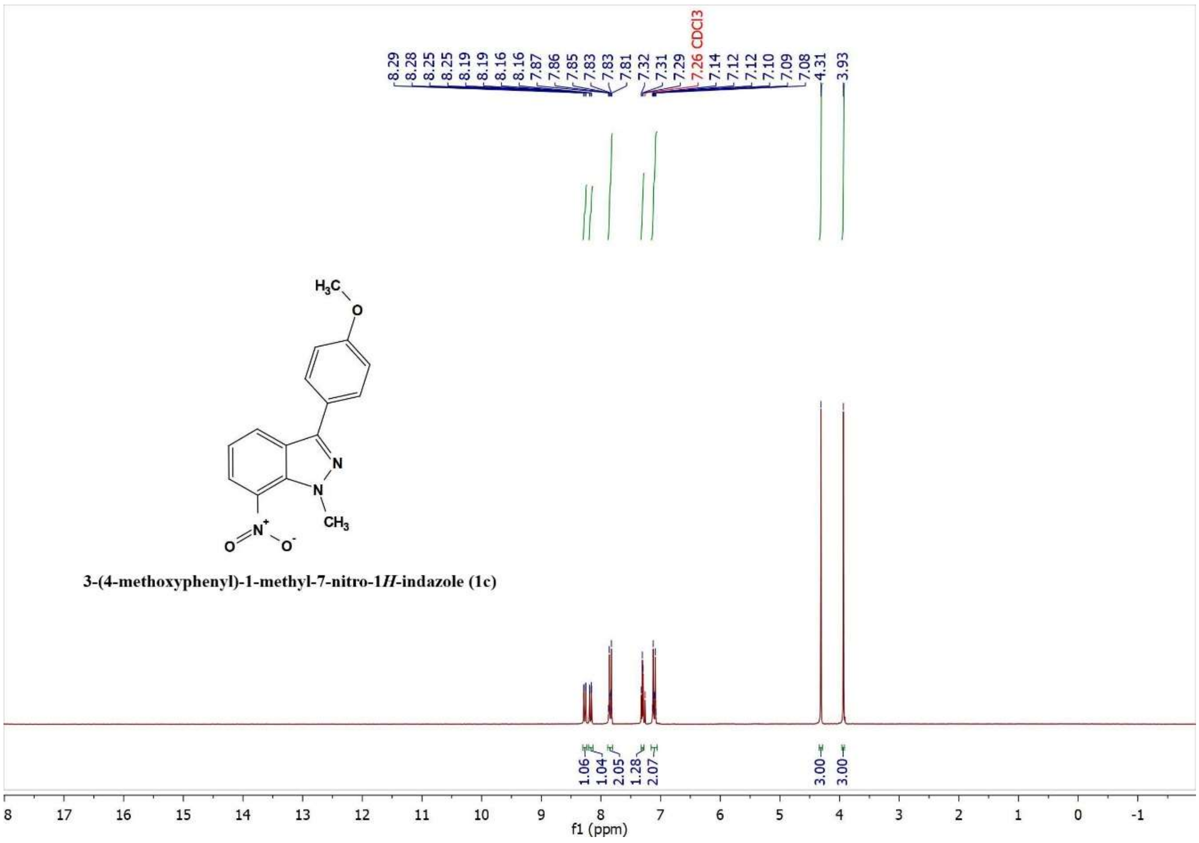

Figure 3 : *^{1}H NMR spectrum (400 MHz, CDCl$_3$) of compound 1c*

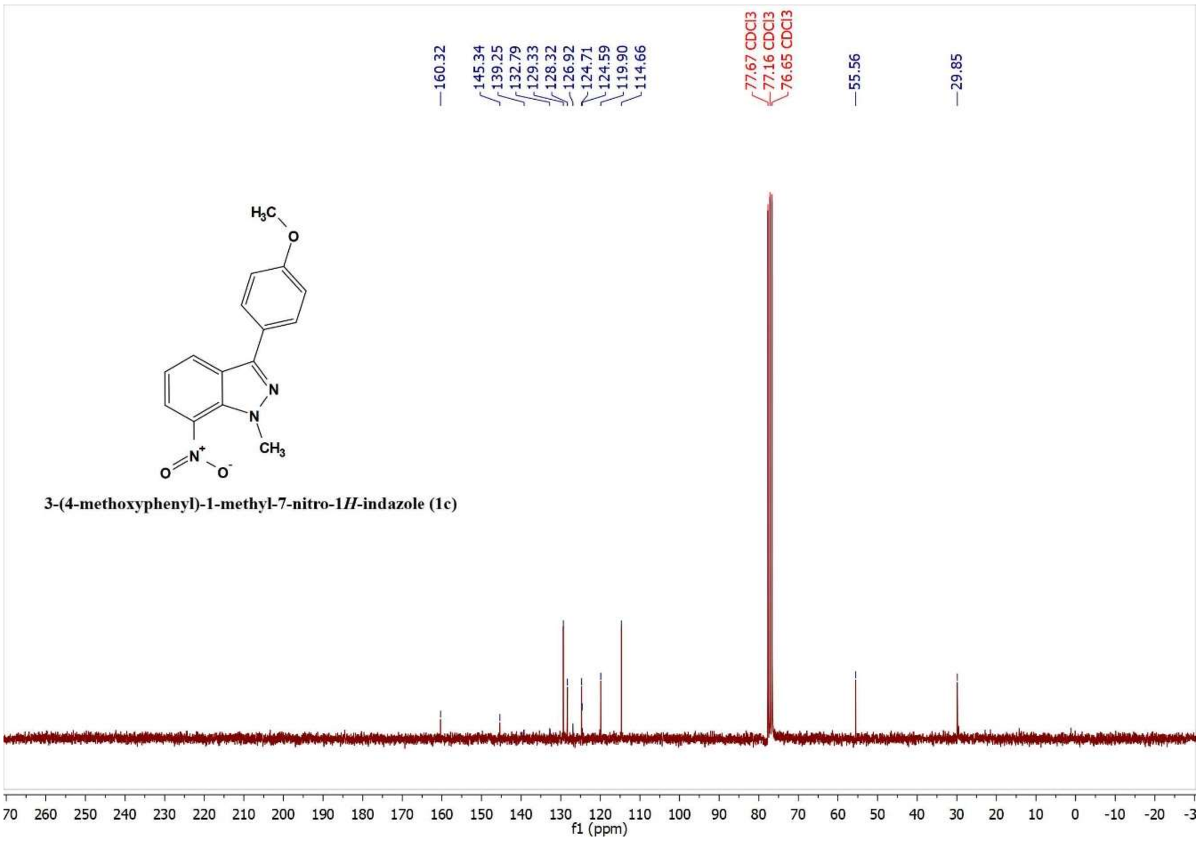

Figure 4 : *^{13}C NMR spectrum (101 MHz, CDCl$_3$) of compound 1c*

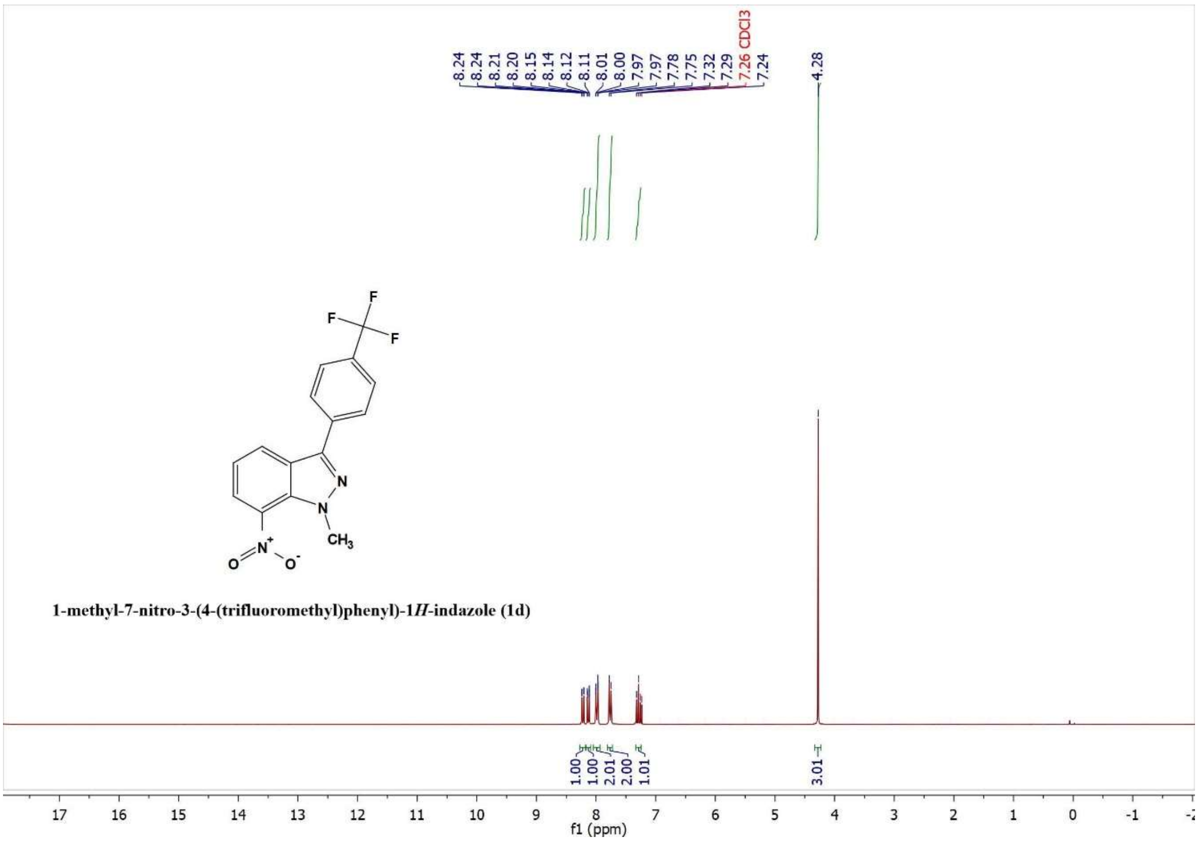

Figure 5 *: ^{1}H NMR spectrum (400 MHz, $CDCl_3$) of compound 1d*

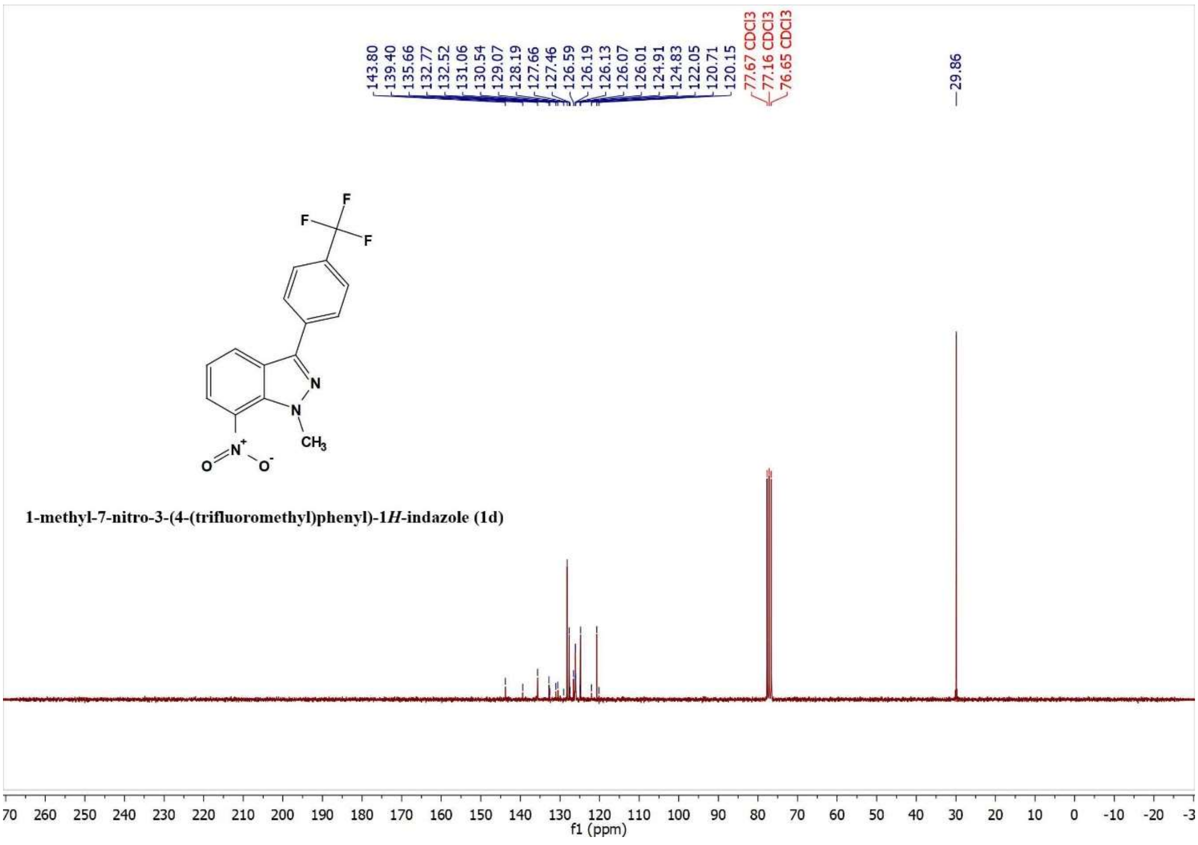

Figure 6 *: ^{13}C NMR spectrum (101 MHz, $CDCl_3$) of compound 1d*

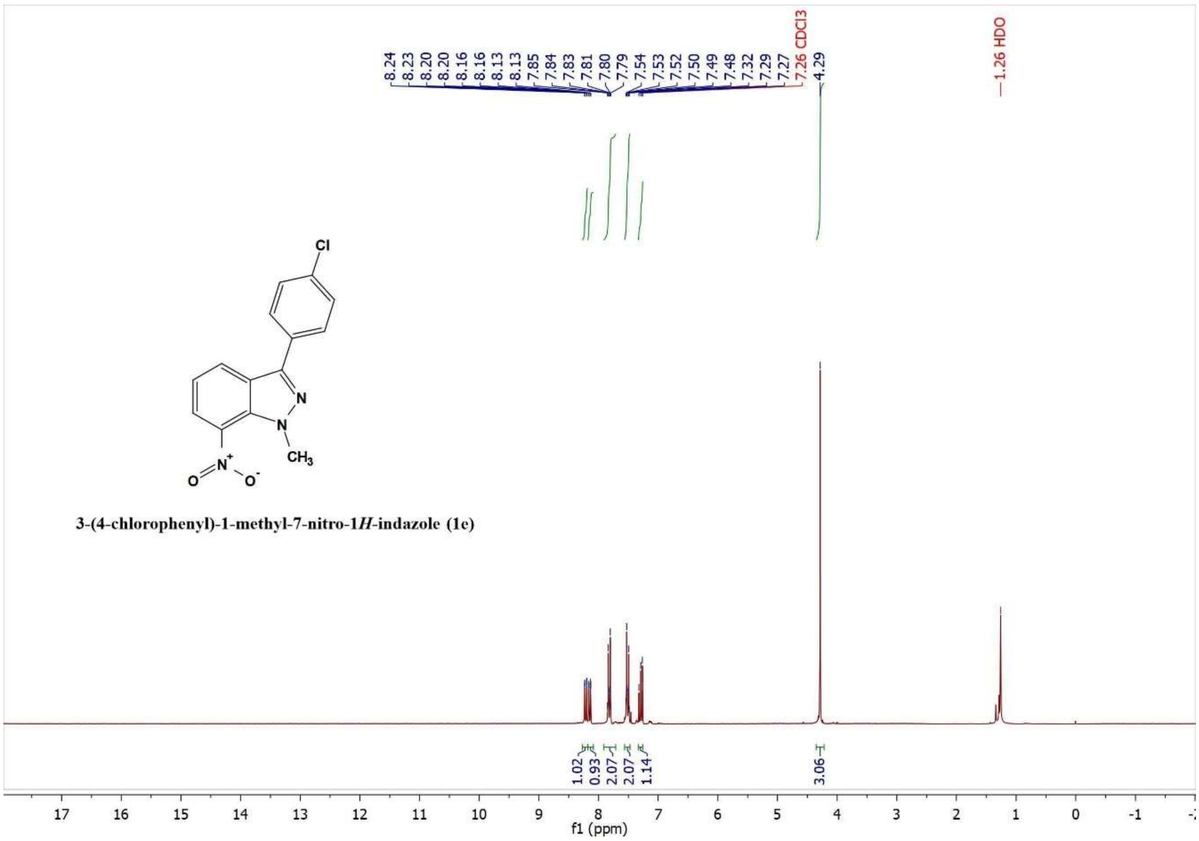

Figure 7 *: ^{1}H NMR spectrum (400 MHz, $CDCl_3$) of compound 1e*

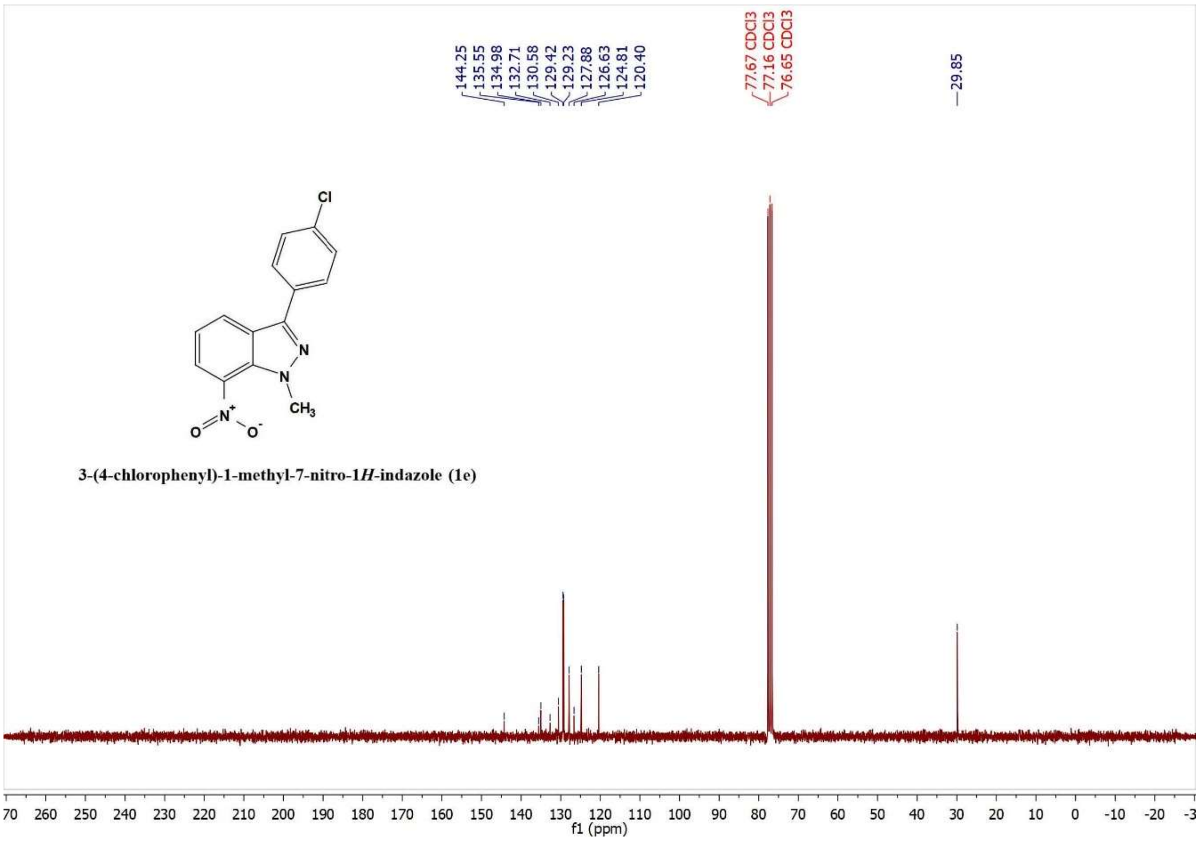

Figure 8 *: ^{13}C NMR spectrum (101 MHz, $CDCl_3$) of compound 1d*

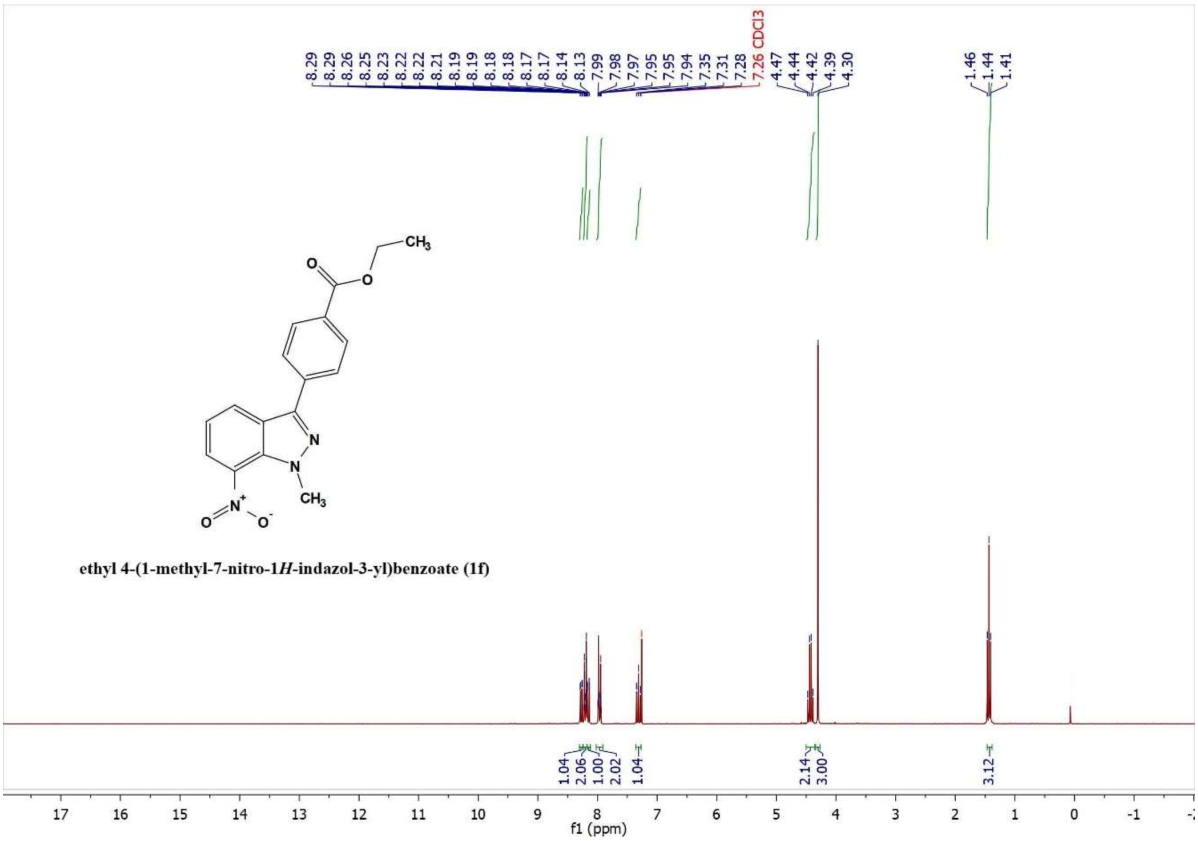

Figure 9 *: ^{1}H NMR spectrum (400 MHz, CDCl₃) of compound 1f*

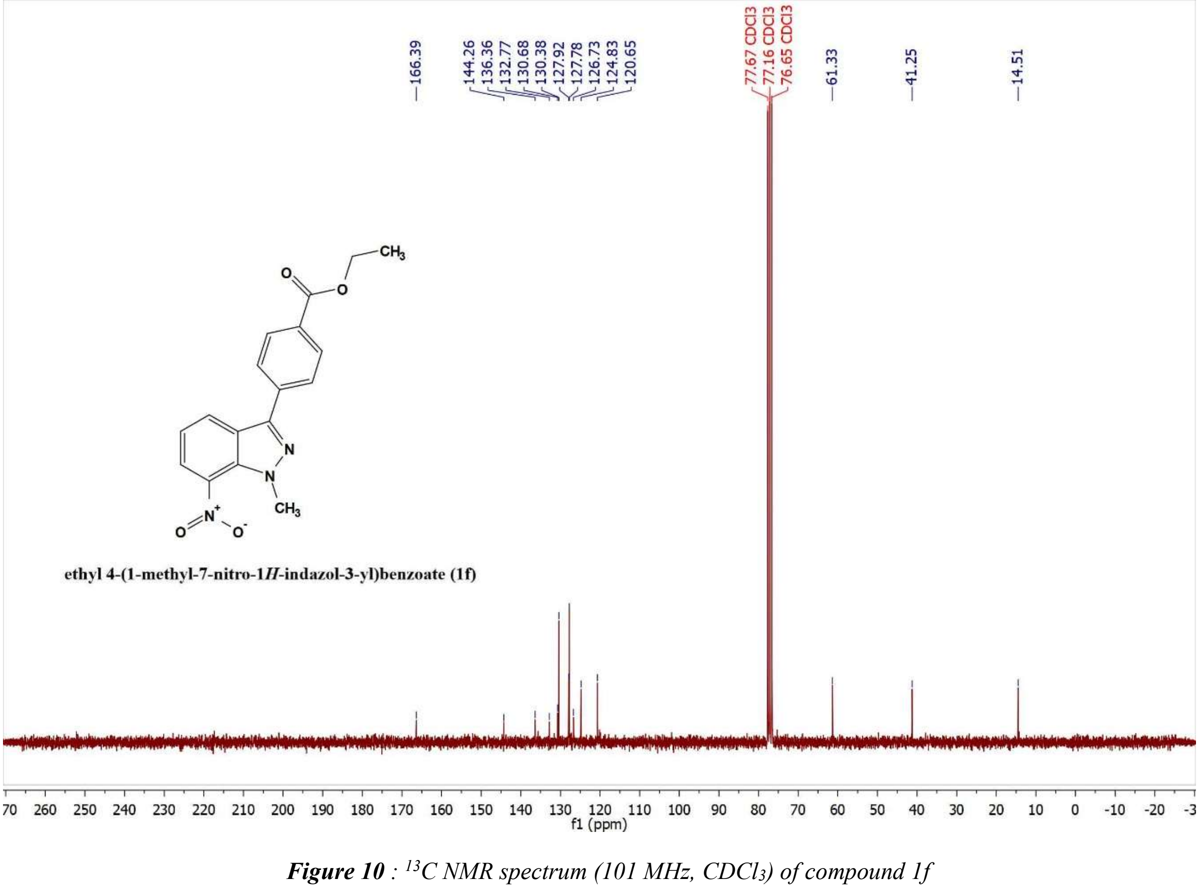

Figure 10 *: ^{13}C NMR spectrum (101 MHz, CDCl₃) of compound 1f*

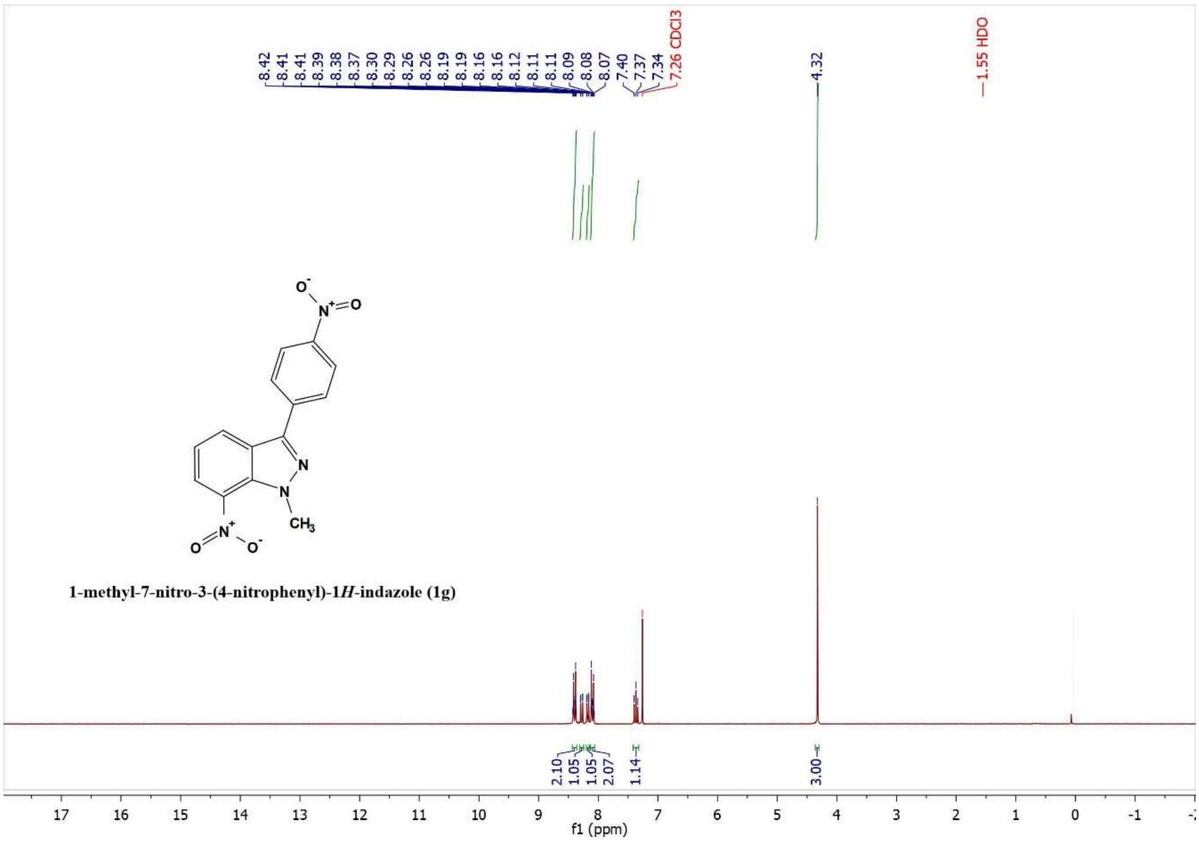

Figure 11 *: ^{1}H NMR spectrum (400 MHz, $CDCl_3$) of compound 1g*

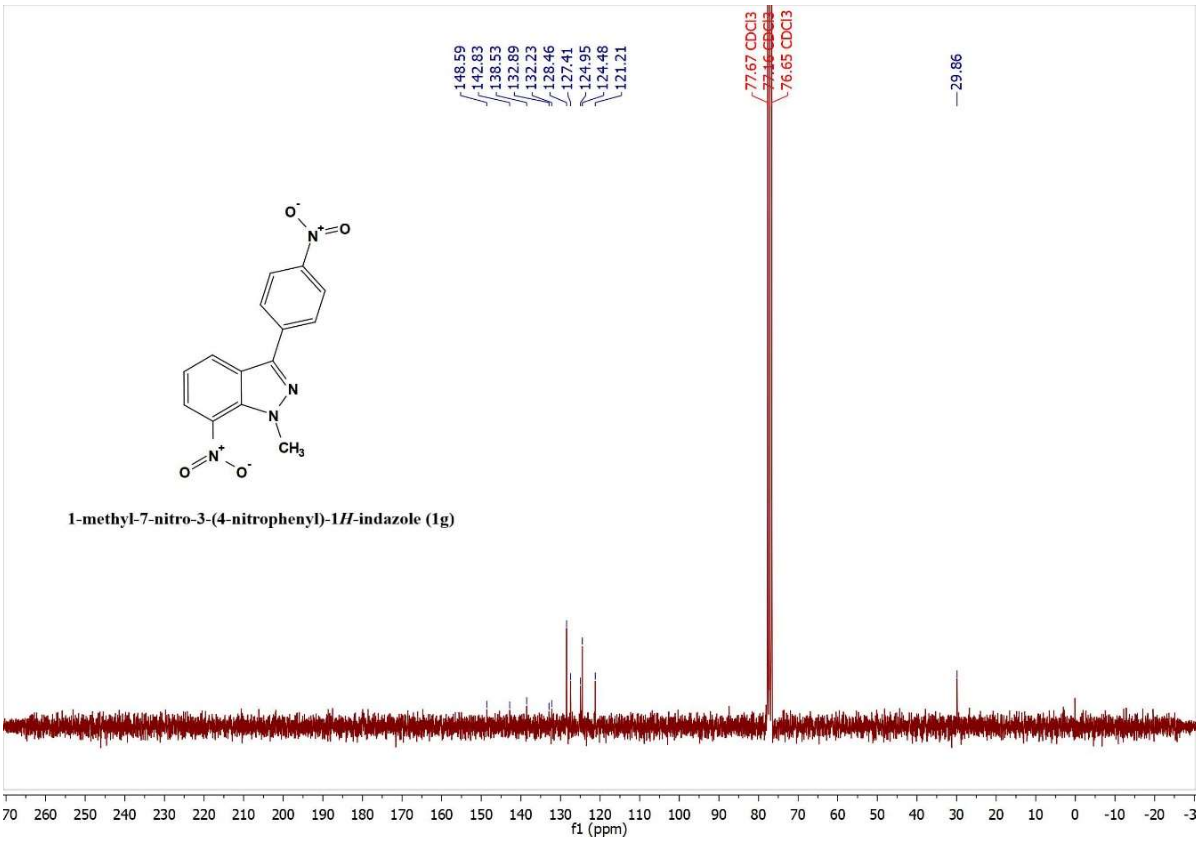

Figure 12 *: ^{13}C NMR spectrum (101 MHz, $CDCl_3$) of compound 1g*

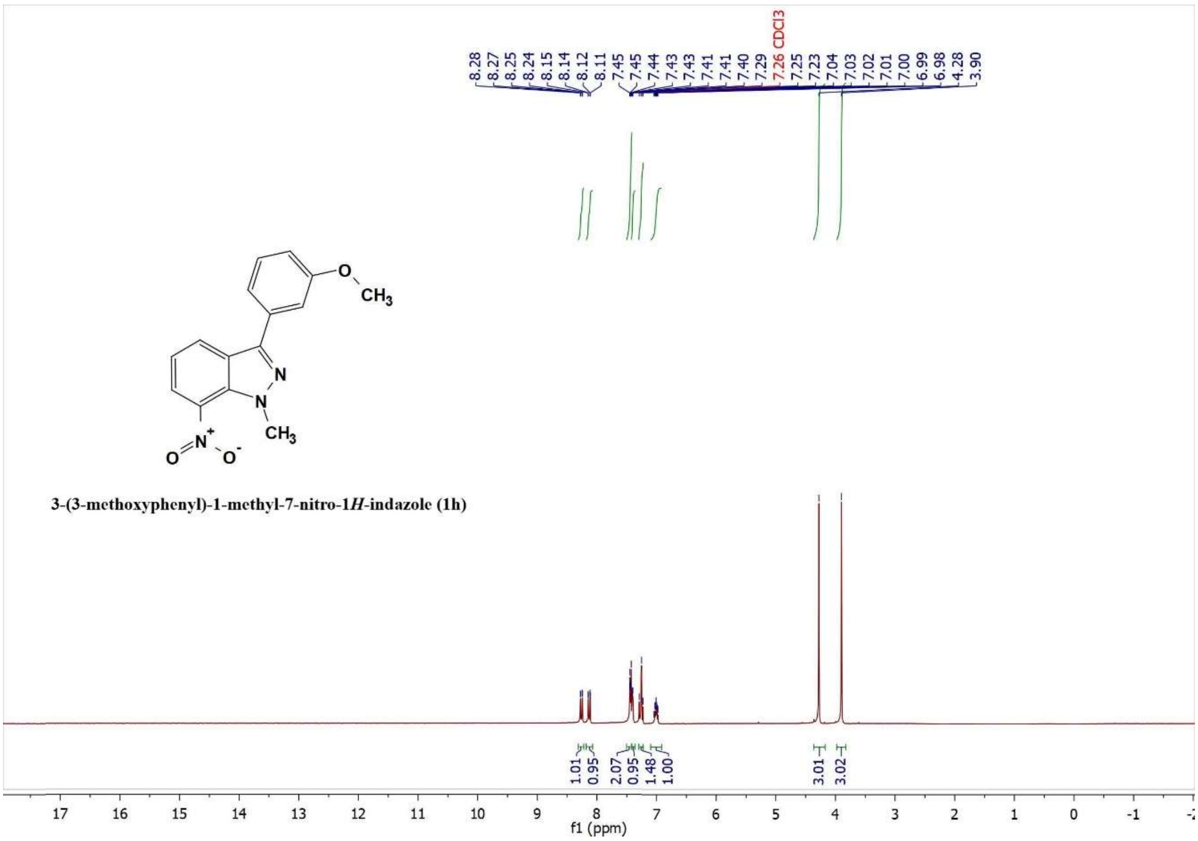

Figure 13 *: ^{1}H NMR spectrum (400 MHz, $CDCl_3$) of compound 1h*

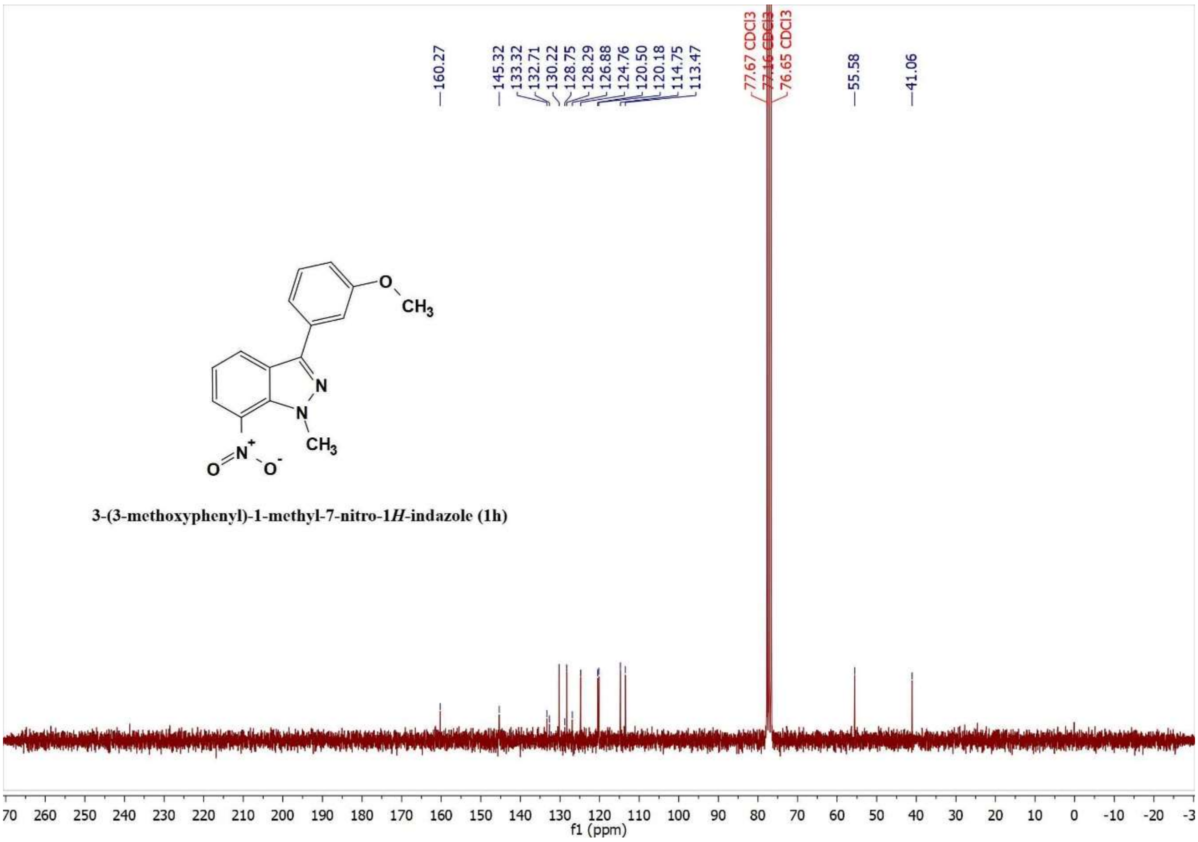

Figure 14 *: ^{13}C NMR spectrum (101 MHz, $CDCl_3$) of compound 1h*

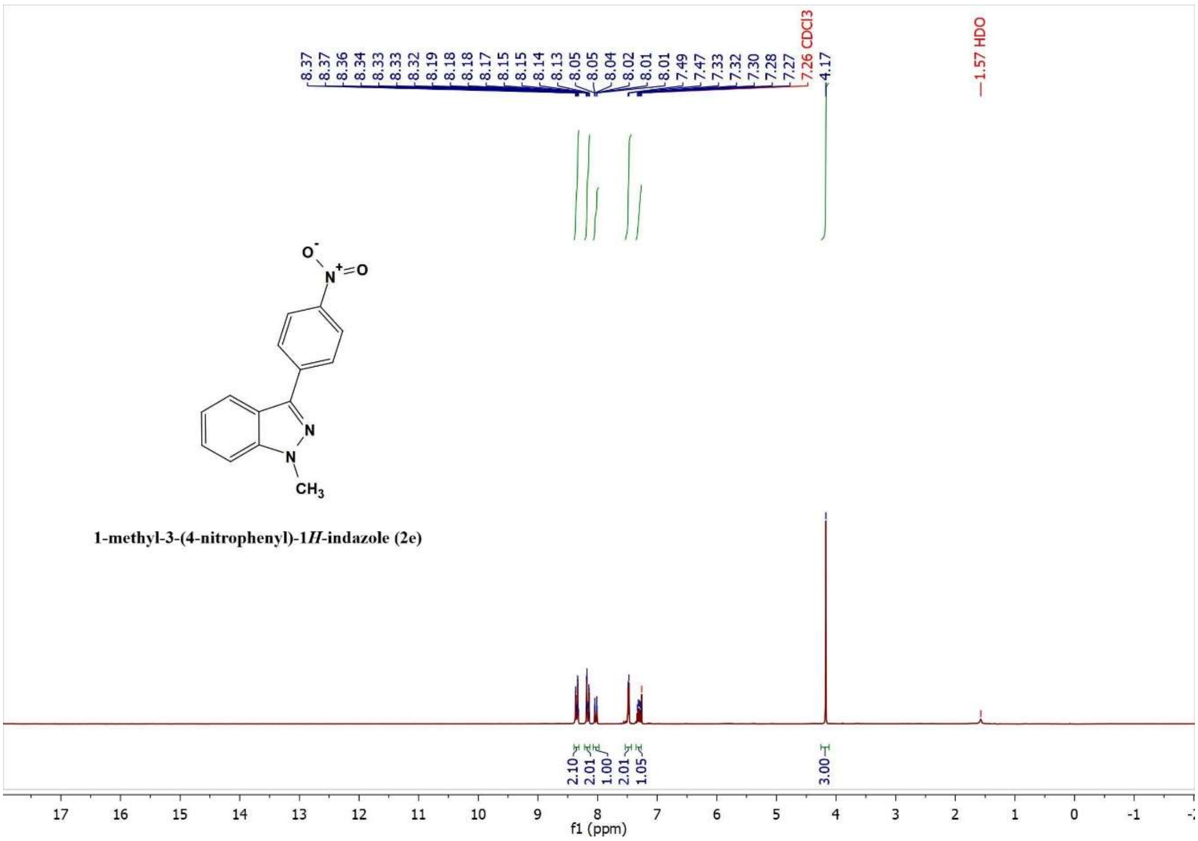

Figure 15 *: ^{1}H NMR spectrum (400 MHz, $CDCl_3$) of compound 2e*

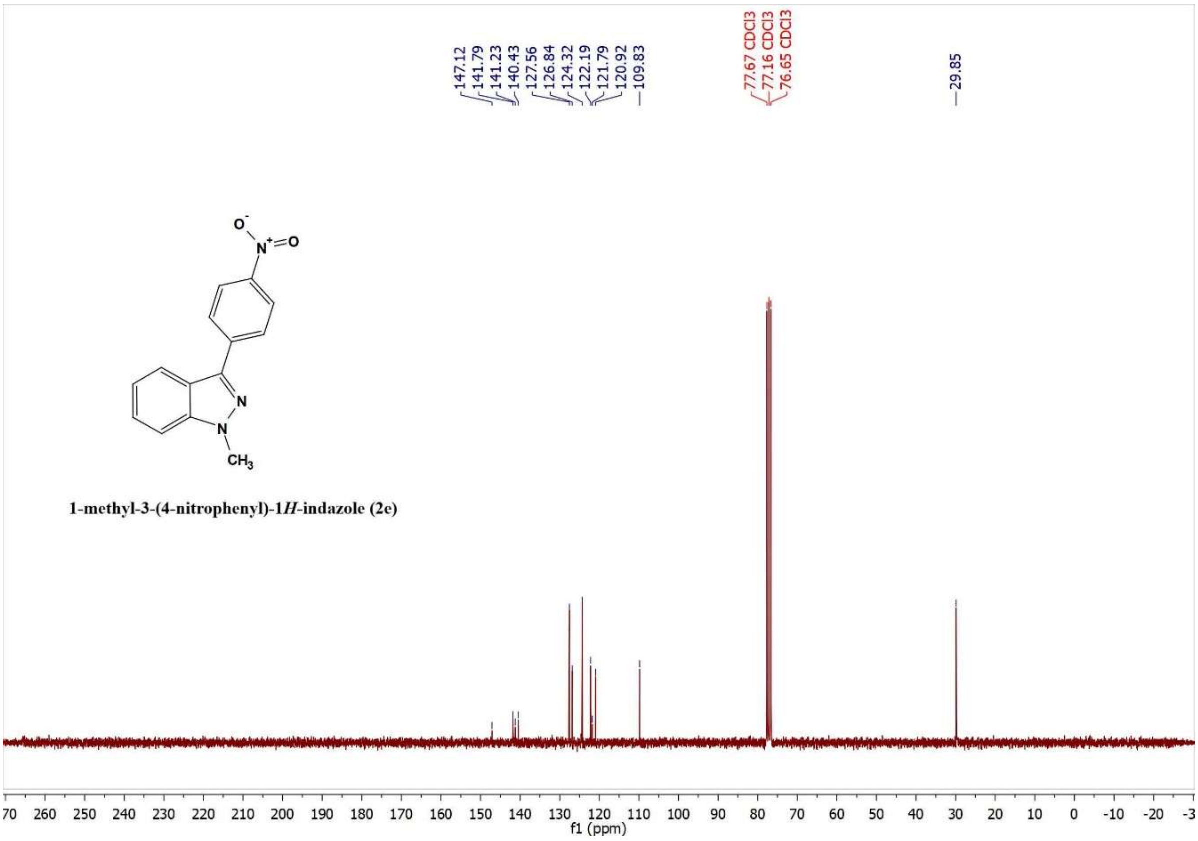

Figure 16 *: ^{13}C NMR spectrum (101 MHz, $CDCl_3$) of compound 2e*

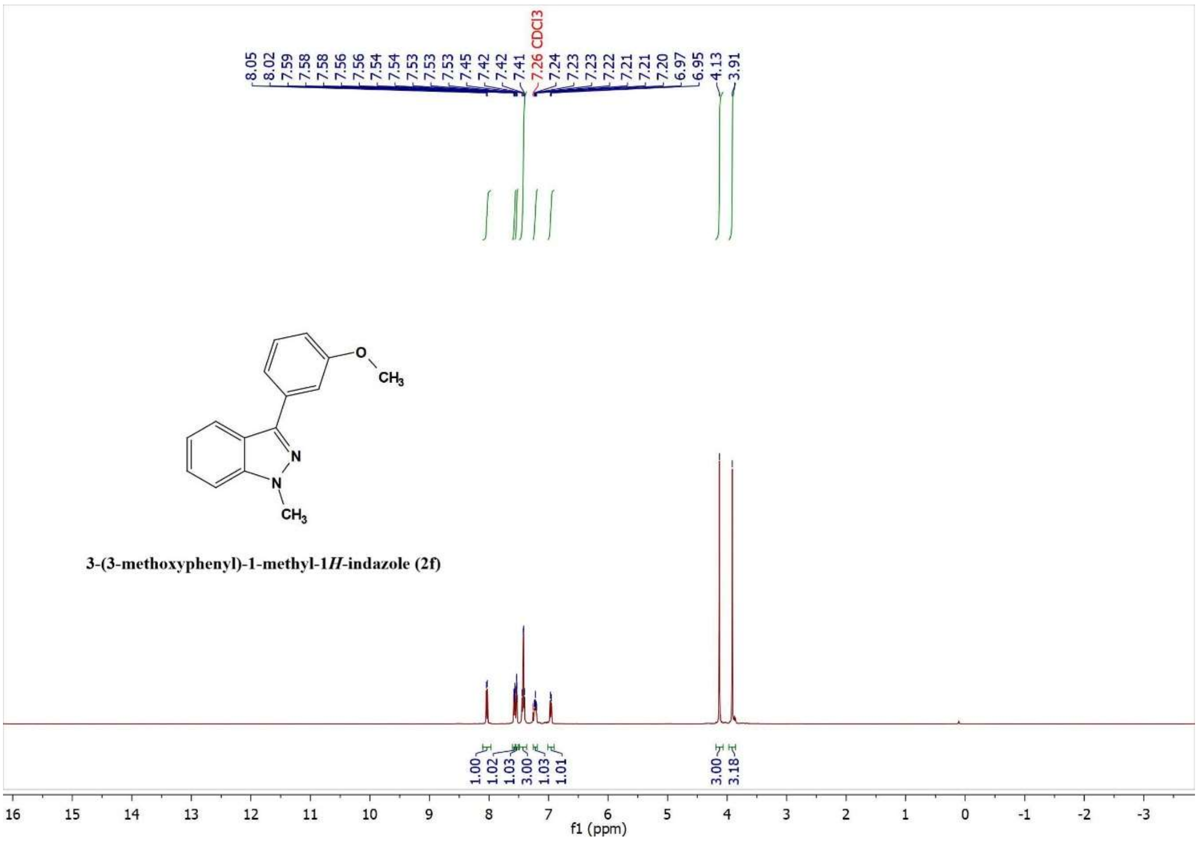

Figure 17 : *^{1}H NMR spectrum (400 MHz, $CDCl_3$) of compound 2f*

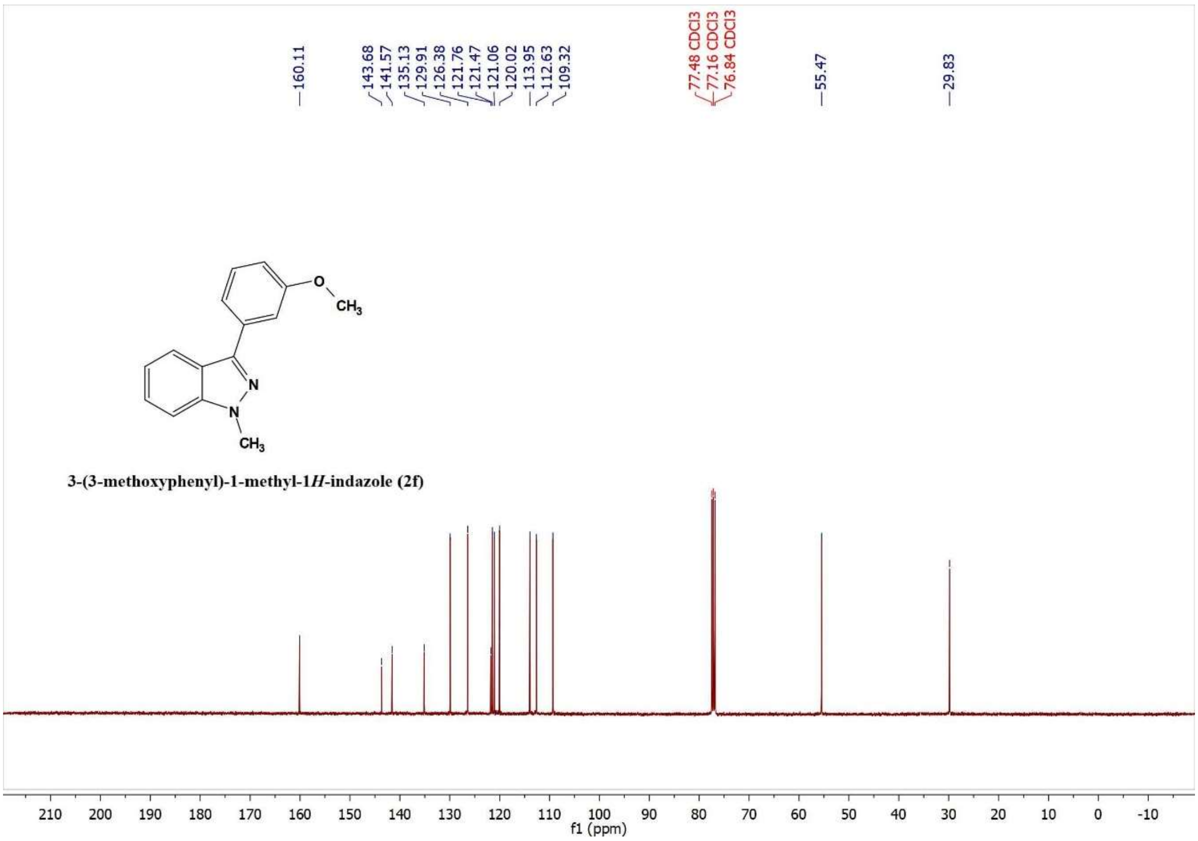

Figure 18 *: ^{13}C NMR spectrum (101 MHz, $CDCl_3$) of compound 2f*

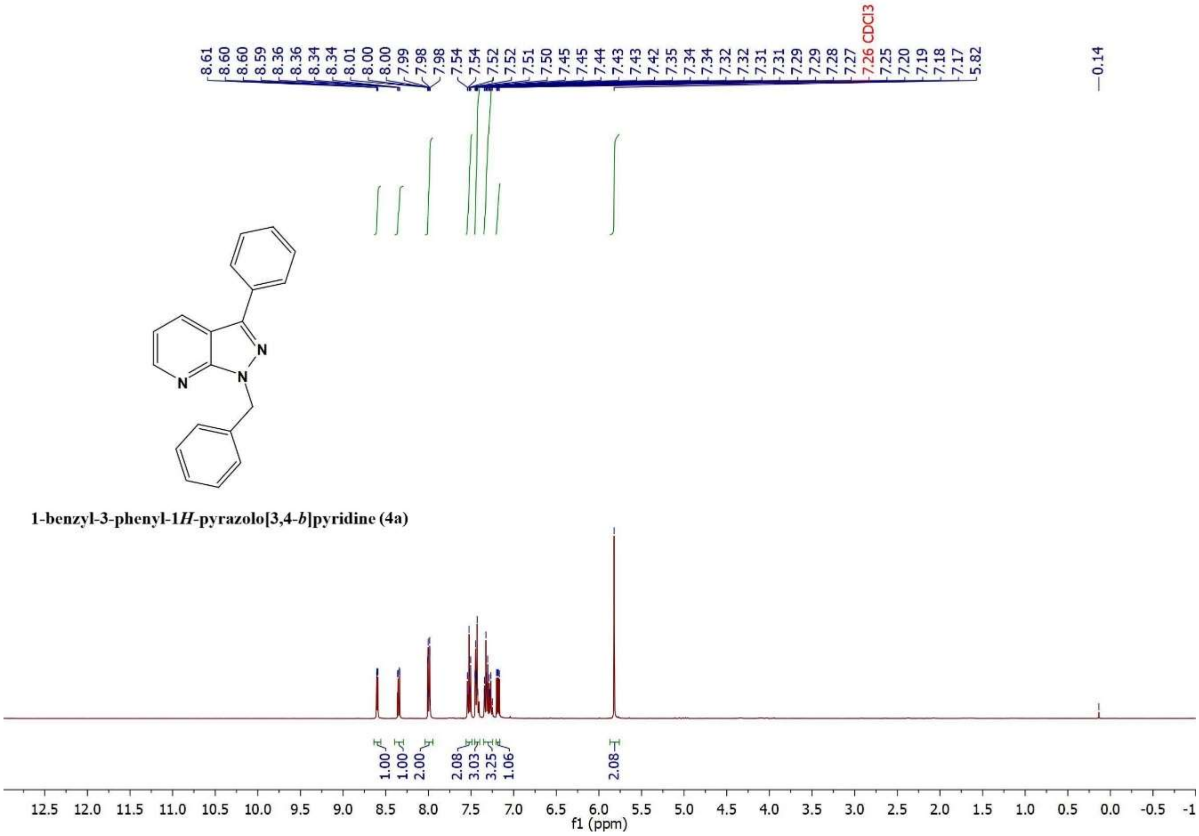

Figure 19 : *¹H NMR spectrum (400 MHz, CDCl₃) of compound 4a*

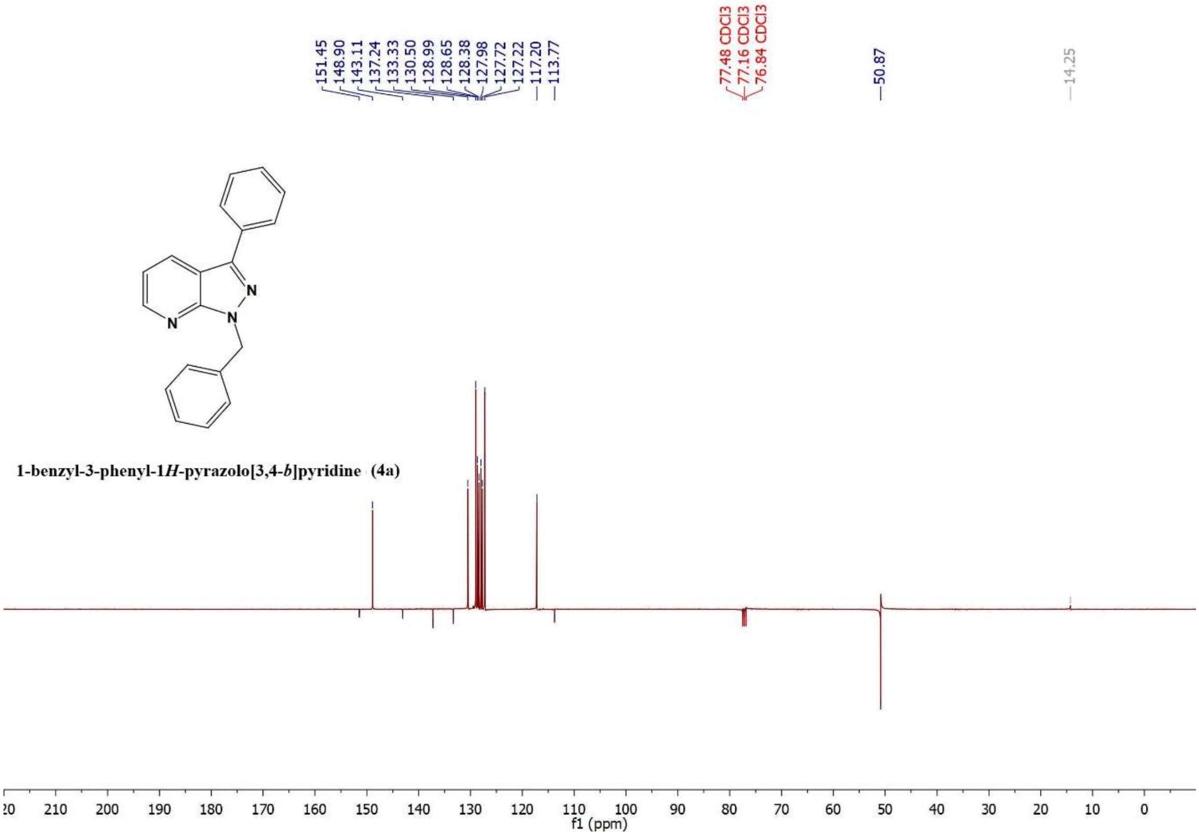

Figure 20 *: ¹³C Jmod NMR spectrum (101 MHz, CDCl₃) of compound 4a*

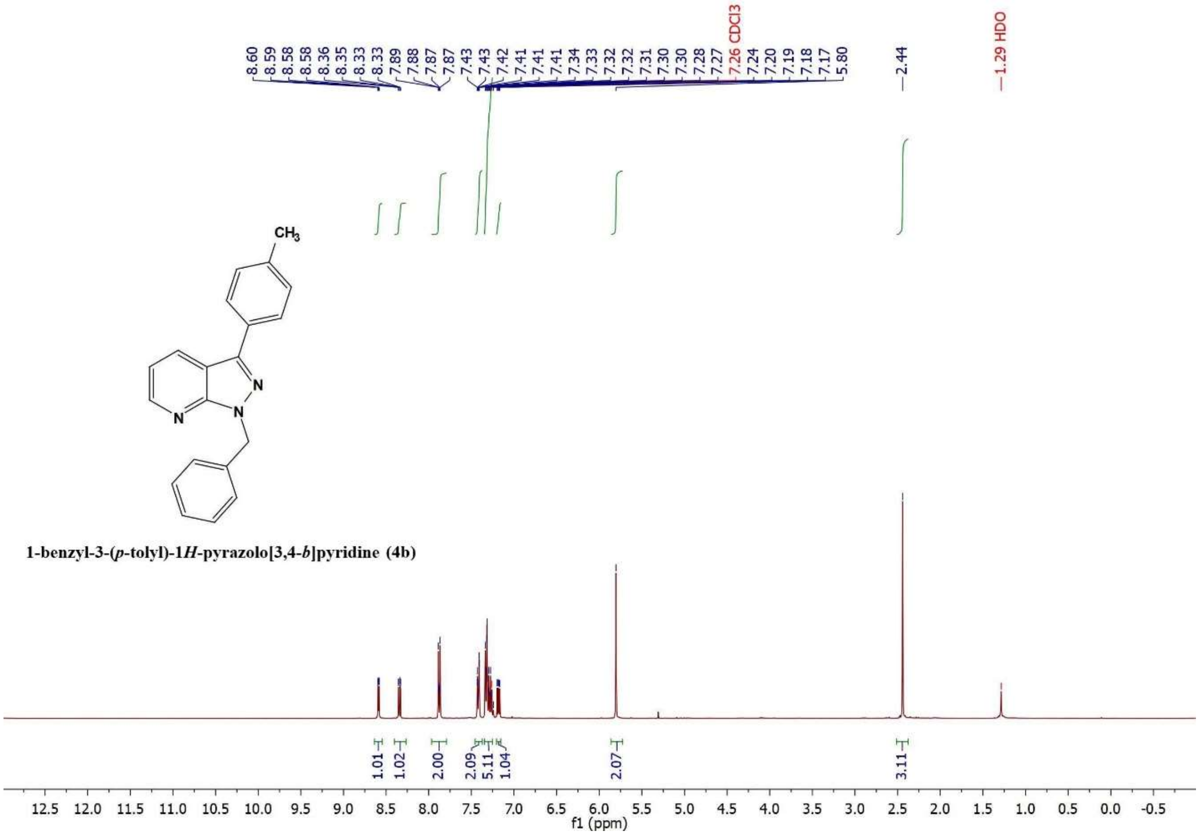

Figure 21 : *^{1}H NMR spectrum (400 MHz, $CDCl_3$) of compound 4b*

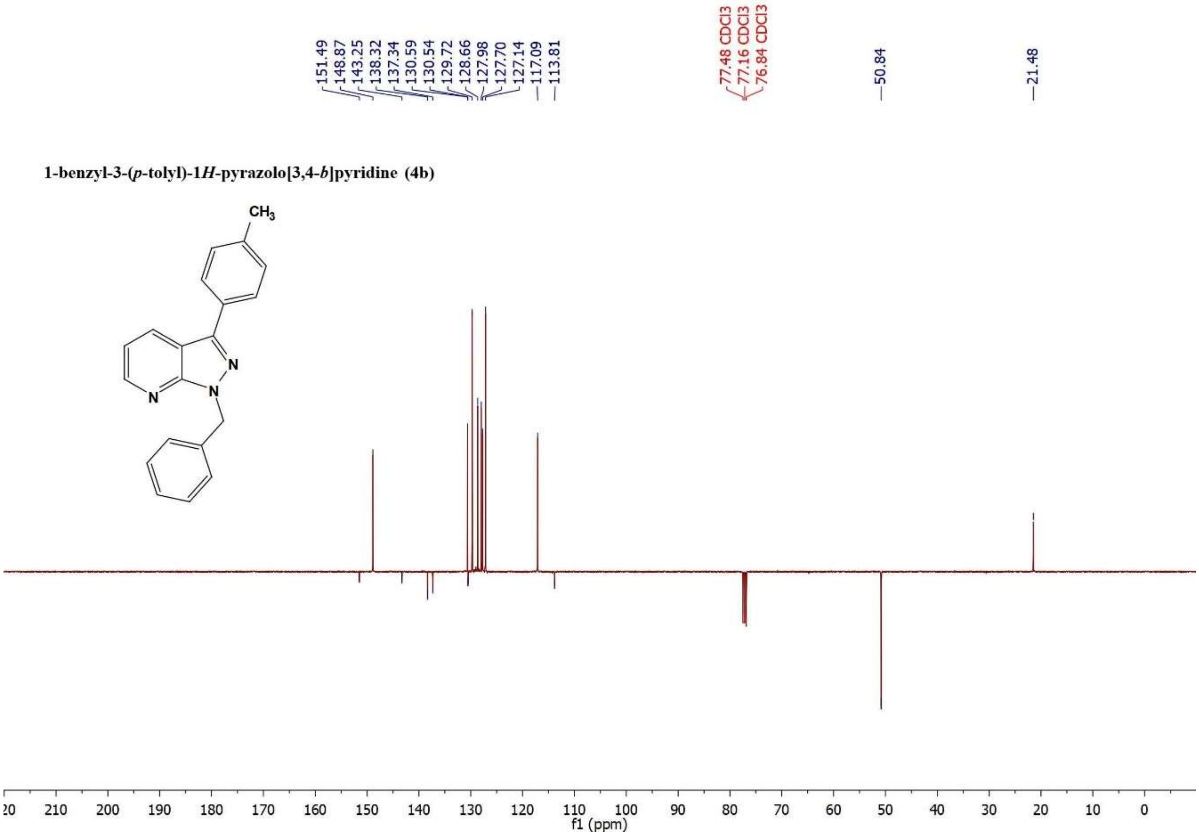

Figure 22 : *^{13}C Jmod NMR spectrum (101 MHz, $CDCl_3$) of compound 4b*

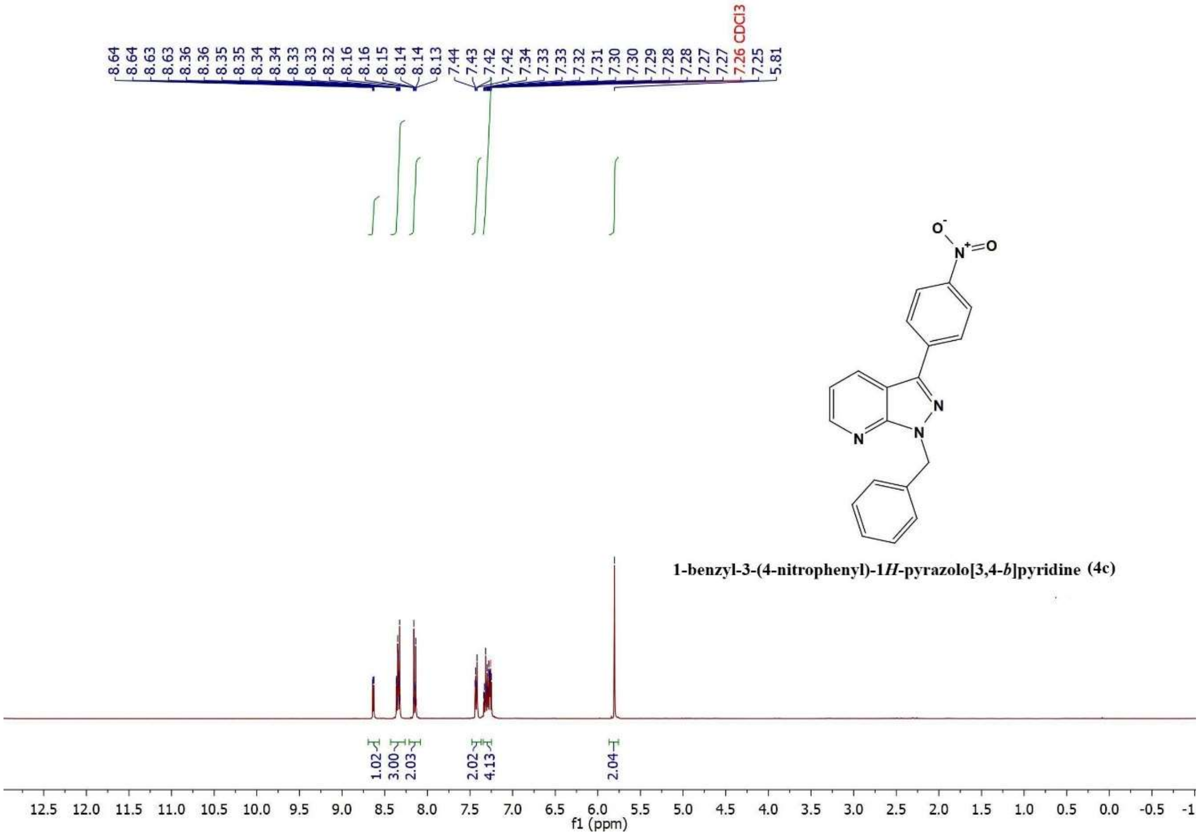

Figure 23 : *^{1}H NMR spectrum (400 MHz, CDCl₃) of compound 4c*

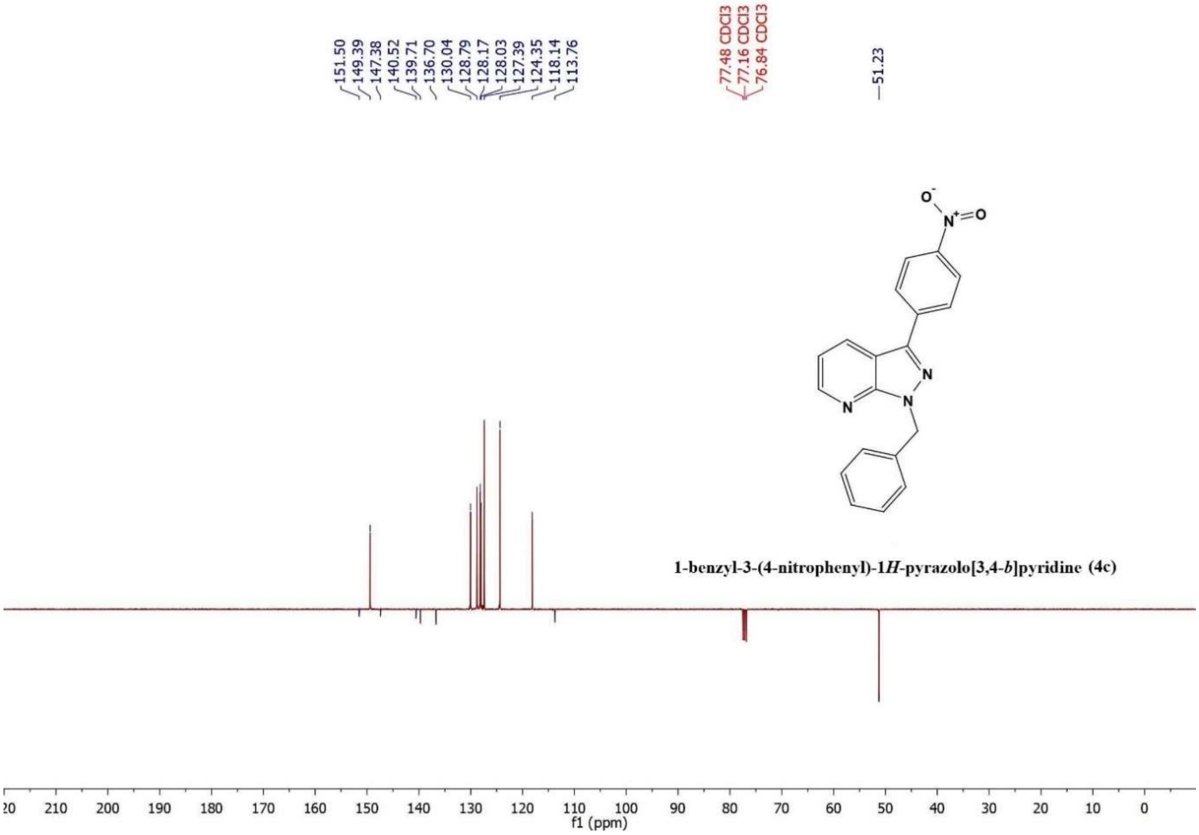

Figure 24 : *^{13}C Jmod NMR spectrum (101 MHz, CDCl₃) of compound 4c*

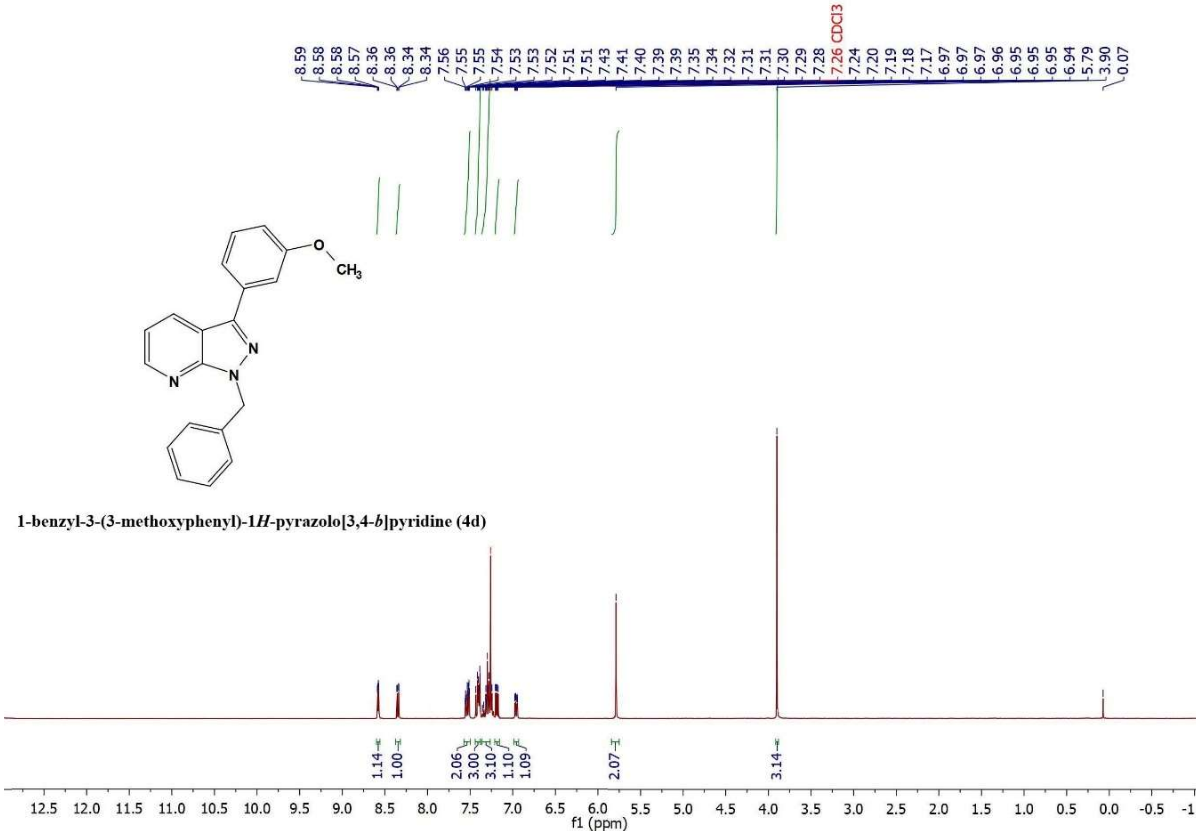

Figure 25 *: ^{1}H NMR spectrum (400 MHz, CDCl₃) of compound 4d*

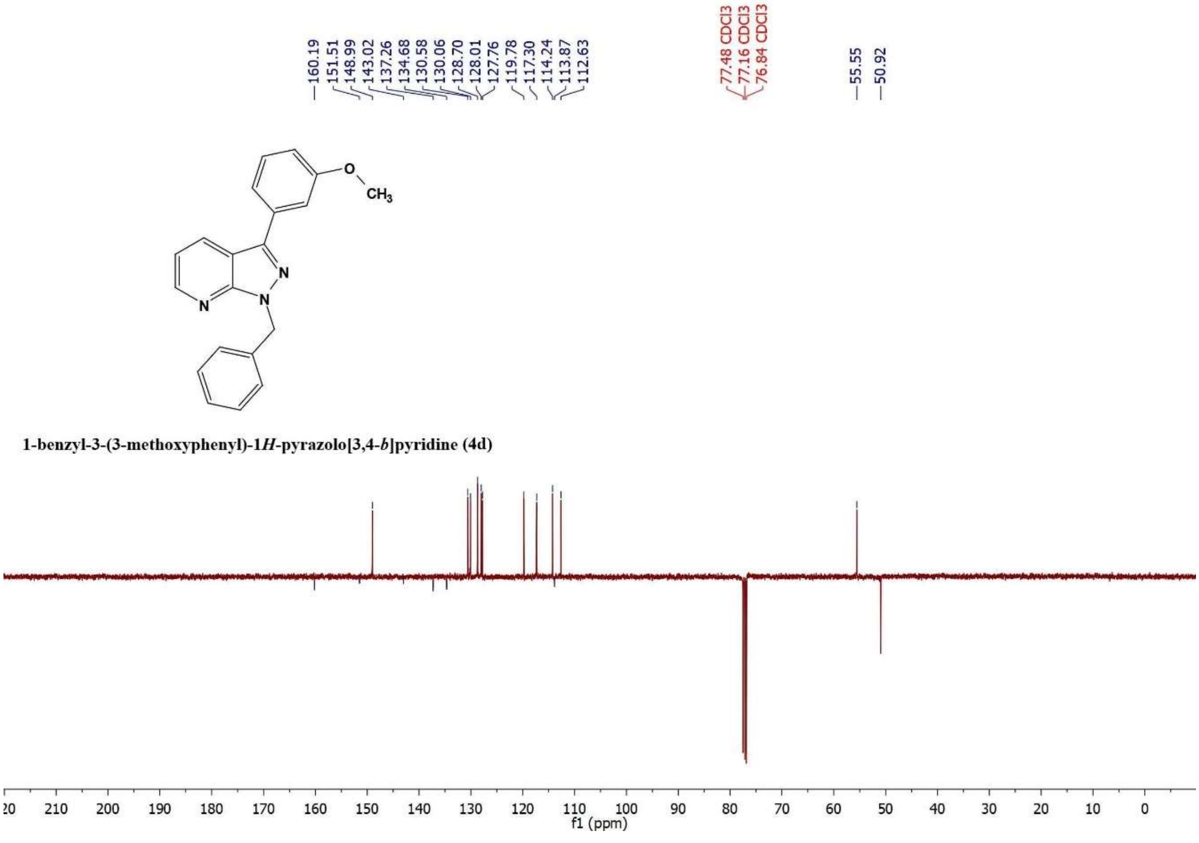

Figure 26 : *^{13}C Jmod NMR spectrum (101 MHz, CDCl₃) of compound 4d*
